# An Integrated—Omics/Chemistry Approach Unravels Enzymatic and Spontaneous Steps to Form Flavoalkaloidal Nudicaulin Pigments in Flowers of *Papaver nudicaule* L.

**DOI:** 10.3390/ijms22084129

**Published:** 2021-04-16

**Authors:** Bettina Dudek, Anne-Christin Warskulat, Heiko Vogel, Natalie Wielsch, Riya Christina Menezes, Yvonne Hupfer, Christian Paetz, Steffi Gebauer-Jung, Aleš Svatoš, Bernd Schneider

**Affiliations:** Max Planck Institute for Chemical Ecology, Hans-Knöll-Straße 8, D-07745 Jena, Germany; bdudek@ice.mpg.de (B.D.); anne.warskulat@yahoo.de (A.-C.W.); hvogel@ice.mpg.de (H.V.); nwielsch@ice.mpg.de (N.W.); rmenezes@ice.mpg.de (R.C.M.); yhupfer@ice.mpg.de (Y.H.); cpaetz@ice.mpg.de (C.P.); gebauer-jung@ice.mpg.de (S.G.-J.); svatos@ice.mpg.de (A.S.)

**Keywords:** *Papaver nudicaule*, nudicaulin biosynthesis, flower pigments, indole alkaloids, indole-3-glycerol-phosphate lyase, transcriptomics, proteomics, metabolomics, free indole, pelargonidin glycosides

## Abstract

Flower colour is an important trait for plants to attract pollinators and ensure their reproductive success. Among yellow flower pigments, the nudicaulins in *Papaver nudicaule* L. (Iceland poppy) are unique due to their rarity and unparalleled flavoalkaloid structure. Nudicaulins are derived from pelargonidin glycoside and indole, products of the flavonoid and indole/tryptophan biosynthetic pathway, respectively. To gain insight into the molecular and chemical basis of nudicaulin biosynthesis, we combined transcriptome, differential gel electrophoresis (DIGE)-based proteome, and ultra-performance liquid chromatography–high resolution mass spectrometry (UPLC-HRMS)-based metabolome data of *P. nudicaule* petals with chemical investigations. We identified candidate genes and proteins for all biosynthetic steps as well as some key metabolites across five stages of petal development. Candidate genes of amino acid biosynthesis showed a relatively stable expression throughout petal development, whereas most candidate genes of flavonoid biosynthesis showed increasing expression during development followed by downregulation in the final stage. Notably, gene candidates of indole-3-glycerol-phosphate lyase (IGL), sharing characteristic sequence motifs with known plant IGL genes, were co-expressed with flavonoid biosynthesis genes, and are probably providing free indole. The fusion of indole with pelargonidin glycosides was retraced synthetically and promoted by high precursor concentrations, an excess of indole, and a specific glycosylation pattern of pelargonidin. Thus, nudicaulin biosynthesis combines the enzymatic steps of two different pathways with a spontaneous fusion of indole and pelargonidin glycoside under precisely tuned reaction conditions.

## 1. Introduction

**Figure 1:**
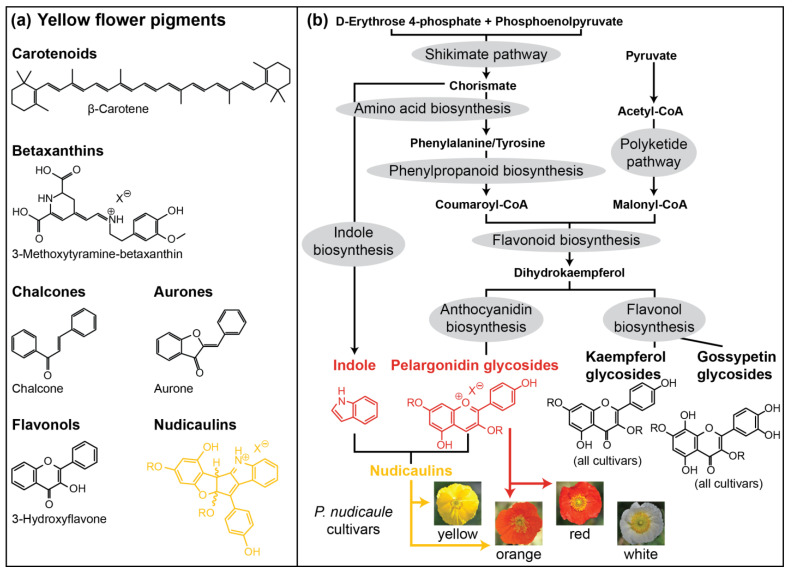
(**a**) Prominent representatives of yellow flower pigment classes. (**b**) Suggested biosynthetic pathways leading to pigment formation in *P. nudicaule* petals [27]. UV absorbing kaempferol glycosides and yellow gossypetin glycosides (basal petal part) are present in all cultivars. Red pelargonidin glycosides are present in orange and red flowers. Yellow nudicaulins are present in yellow and orange flowers. R represents glycosyl unit or proton, X^θ^ represents counterion of unspecified identity.

Flower appearance is one of the most important communication tools of plants, especially to attract pollinators [1,2]. Flower colour is determined by pigments absorbing light of certain wavelengths and by the scattering structures of the petal surface and interior [3,4,5,6,7]. Flower pigments can additionally serve other functions, e.g., in protecting tissue from UV damage, drought, and cold [8,9,10,11,12,13,14]. Most pigments belong to only few structural classes, particularly to water soluble flavonoids and water insoluble carotenoids [15,16]. Approximately one third of all flowering plants in different regions of the world have yellow flowers [17]. Furthermore, yellow is also the colour of most pollen, and is thus very attractive to some insects, e.g., flies and bumblebees [18,19,20]. Most yellow to orange flowers are coloured by carotenoids [15]. Besides the carotenoids, betaxanthins as well as some flavonoidal compounds with aurone, chalcone, and flavonol structures are yellow (Figure 1a) [21]. Whereas betaxanthins only occur in the order Caryophyllales, the yellow flavonoids are more widely distributed (e.g., in Asterales, Laminales, Ericales, and Malvales) [21]. In many cases, yellow colour is combined with reflection in the UV range [22], which can also be perceived by many insects, including bees [23,24]. UV reflection is usually a result of the absence of UV absorbing pigments [25,26].

**Figure 2:**
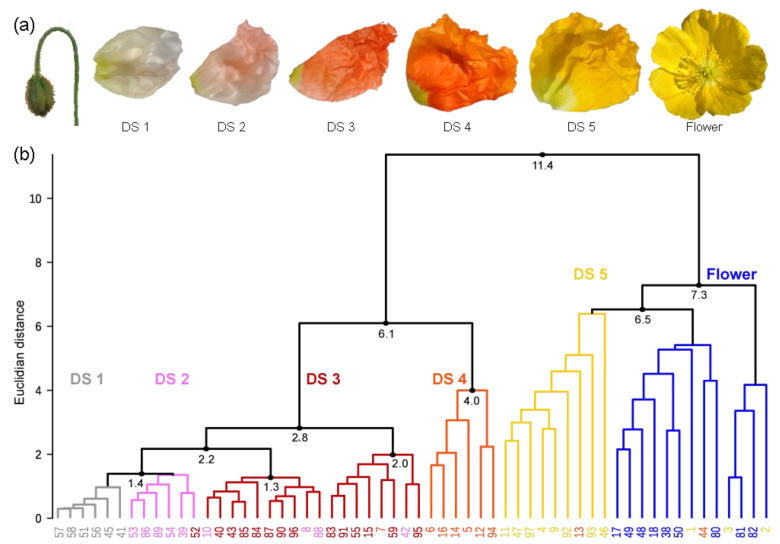
(**a**) Petals from five developmental stages (DSs) inside buds and a flower of the yellow *P. nudicaule* cultivar. (**b**) Dendrogram of the cluster analysis from petal extracts of the yellow *P. nudicaule* cultivar. The sample numbers are coloured according to the respective petal appearance (white—labelled in grey, pale red—labelled in pink, red, orange, yellow, open flowers—labelled in blue), which corresponds to petal age in closed buds. In most cases, petals of the same colour formed clusters, which are labelled according to the predominant petal colour and represent five DSs and the open flower.

One exceptional class of yellow pigments are the nudicaulins (Figure 1a), indole/flavonoid hybrid alkaloids from the apical petal part of yellow and orange flowers of *Papaver nudicaule* L., the Iceland poppy [28,29,30], and two closely related species, *Papaver alpinum* (Alpine poppy) and *Meconopsis cambrica* (Welsh poppy) [31,32]. *P. nudicaule* (Papaveraceae, section Scapiflora) is native in mountain areas, along rivers, on meadows, and on the steppes of Siberia and Mongolia [32,33,34], and has been cultivated in Europe since the 18th century [32]. In nature, yellow flowers are dominant, while white and orange flowers are rare [34]. Nowadays, due to breeding, plants with red and pink flowers are also available. The nudicaulins have a unique glycosylated flavoalkaloid structure, fully characterised after more than 70 years of research in 2013 [35]. The structure consists of an indolic and a polyphenolic part, and free indole and pelargonidin glycosides were identified as the direct precursors by ^13^C labelling experiments. The assembly of nudicaulin from these precursors was mimicked in vivo and in vitro [27,36]. The biosynthetic pathways of indole and pelargonidin are known from other plants—both compounds are derived from the shikimate and aromatic amino acid biosynthesis (Figure 1b). Indole is usually a precursor of tryptophan and, in plants, only occurs in its free form when used in the synthesis of specialised metabolites or when released as a volatile [37,38,39,40]. Pelargonidin glycosides, on the other hand, are red anthocyanin pigments frequently occurring in flowers. They are derived from the flavonoid pathway [41,42], starting with the condensation of *p*-coumaroyl-CoA with three malonyl-CoA units. These initial steps, catalysed by chalcone synthase, a plant-specific polyketide synthase III enzyme [43], are followed by multiple conversions (condensation, isomerisation, oxidation, and reduction) resulting in different flavonoid compounds. Due to the gradual accumulation of red pelargonidin glycosides during flower development, petals of the yellow *P. nudicaule* cultivar change their colour from white over pale red to red in closed buds. Starting with the conversion of pelargonidin glycosides into nudicaulins, the petals turn orange, yellow, and finally, flowers start blooming (Figure 2a) [27]. Petals of orange and red cultivars show a similar colour sequence during development, but changes stop at the respective flower colour.

Although retrobiosynthetic labelling and precursor-directed biosynthetic studies [27,44] have provided knowledge about the key precursors of nudicaulins, the molecular basis of the nudicaulin biosynthesis in *P. nudicaule* flowers, and especially of the formation of free indole, remains to be substantiated. Additionally, the final step—the fusion of indole and pelargonidin glycosides—is not fully understood. As the reaction was mimicked in vitro and in vivo and executed synthetically under acidic conditions [36,45], it could be a spontaneous non-enzymatic step. Unravelling of these questions is aggravated by the fact that *P. nudicaule* is not a model organism and no genomic information is available. Therefore, we chose a multilevel approach based on transcriptome, proteome, and metabolome data, and additional chemical analyses. For time-resolved investigation of petal development, five developmental stages (DSs) were defined according to pigment production. Enzyme candidates involved in nudicaulin biosynthesis were identified at the mRNA level and verified by proteome data. Additionally, key metabolites and products of the flavonoid biosynthesis were detected by mass spectrometry. The final biosynthetic step and its dependence on concentration and glycosylation pattern of the pelargonidin glycoside were investigated in vitro. The results from the yellow flowering *P. nudicaule* cultivar were compared with differently coloured cultivars and ecological implications are discussed.

## 2. Results and Discussion

### 2.1. Definition of Stages in the Petal Development of the Yellow P. nudicaule Cultivar

The investigation of flower development by multiple methods required the definition of distinct DSs, which would enable a reproducible sampling. In the yellow cultivar, petal colour changes during development (Figure 2a) [27], and thus pigment content could reliably discriminate five DSs. A high performance liquid chromatography—photo diode array (HPLC-PDA) approach, which is sensitive for coloured compounds, was chosen, and data were subjected to a cluster analysis to identify similarity between samples. The 58 buds and flowers, harvested at random time points in their development, were grouped into two main clusters and several subclusters (Figure 2b). The clusters are a good representation of the observed colour change from white over pale red, red, and orange to yellow during the maturation of the tissue. Yellow petals harvested shortly before and after anthesis formed one main cluster. In this, one subcluster was dominated by flowers before anthesis, one by flowers after anthesis, and one was mixed. Several samples were clustered into the wrong subcluster, and the distance between the single samples was, on average, slightly larger than in case of the other subclusters. This indicated that yellow petals show a higher variability than petals of a different colour. All other DSs were contained in the second main cluster, with most orange petals clearly separated from the others. Red, pale red, and white petals formed one big subcluster, wherein white and red ones were clearly distinguished from each other. In the case of pale red, some petals were more similar to the white and some to the red petals. Pale red may therefore be a transition state between white and red.

Although some flowers (e.g., no. 7, 13, 42, 44, 52, 54) were found in different clusters than expected (Figure 2b), a classification of the development of yellow *P. nudicaule* flowers into stages according to their colouring is generally possible. For further analyses, we defined the following DSs: DS 1—white, DS 2—pale red, DS 3—red, DS 4—orange, and DS 5—yellow (before anthesis). Yellow petals of blooming flowers were not considered because the nudicaulin biosynthesis was already completed in that stage and thus the complexity of the dataset could be reduced.

### 2.2. Transcriptome, Proteome, and Metabolome Analysis of the Nudicaulin Biosynthesis

**Figure 3:**
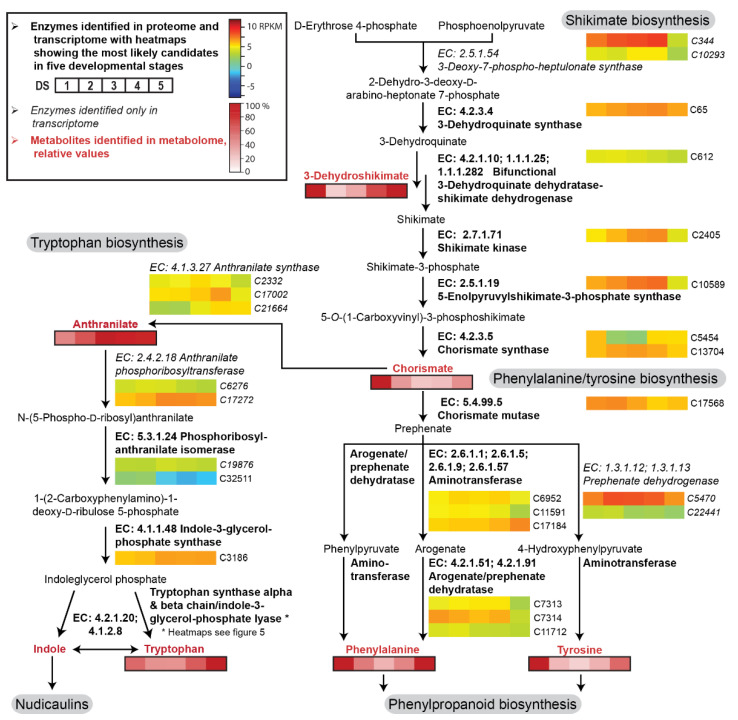
Enzyme candidates and metabolites involved in the shikimate, tryptophan, and phenylalanine biosynthetic pathway in five DSs of the petals of yellow *P. nudicaule* flowers. Heat maps show relative gene expression levels based on log_2_-transformed RPKM values (reads per kilobase million, blue represents weakly expressed genes, and red represents strongly expressed genes, see Table S1). Heat maps of metabolites (black framed boxes) show abundance (mean values, see Table S2) relative to the highest value of each compound.

**Figure 4:**
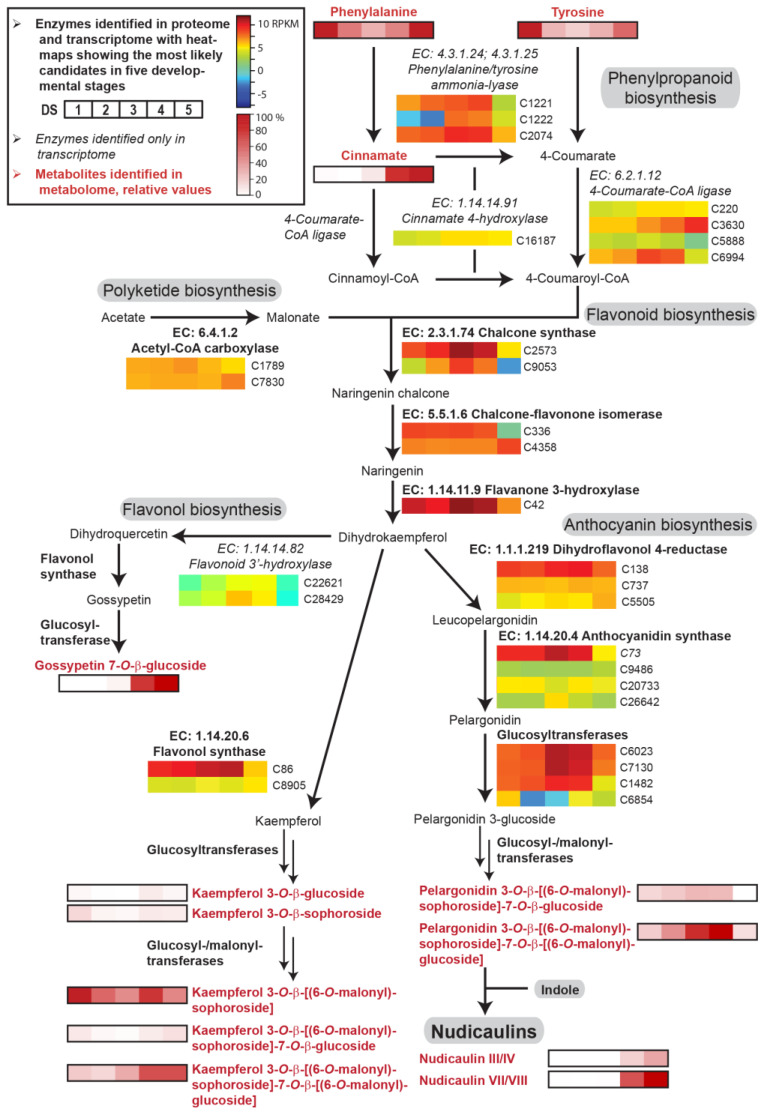
Enzyme candidates and metabolites involved in the phenylpropanoid and flavonoid biosynthetic pathway in five DSs of the petals of yellow *P. nudicaule* flowers. Heat maps show relative gene expression levels based on log2-transformed RPKM values (blue represents weakly expressed genes, and red represents strongly expressed genes, see Table S1). Heat maps of metabolites (black framed boxes) show abundance (mean values, see Table S2) relative to the highest value of each compound or, in the case of kaempferol and pelargonidin glycosides and of nudicaulins, relative to the highest value of all metabolites with the same aglycon. Nudicaulins III/IV bear an 11-*O*-β-d-[(6-*O*-malonyl)sophorosyl] unit and a 7-*O*-β-d-glucosyl unit, nudicaulins VII/VIII bear an 11-*O*-β-d-[(6-*O*-malonyl)sophorosyl] unit and a 7-*O*-β-d-[(6-*O*-malonyl)glucosyl] unit.

As recently reported, nudicaulins are formed from indole and different pelargonidin glycosides [27], whose biosynthetic pathways are well studied in other plants. In *P. nudicaule*, biosynthetic routes that are similar to known pathways are used for the production of nudicaulin precursors. In the present study, we identified candidates for all enzymes potentially involved in nudicaulin biosynthesis at the mRNA and protein level, as well as some key metabolites. Our RNAseq data provided expression levels for the gene candidates over five stages of petal development (Table S1). The most likely candidates were identified by the results of the 2D differential gel electrophoresis (2D-DIGE), which confirmed the presence of most of the respective enzymes (Figure 3 and Figure 4). Unfortunately, the quantitative information from the 2D-DIGE could not be used for comparison, because insufficient separation yielded several proteins per spot.

The shikimate pathway starts with the fusion of d-erythrose 4-phosphate and phosphoenolpyruvate to form 2-dehydro-3-deoxy-d-arabino-heptonate 7-phosphate. This intermediate is converted during several subsequent enzyme reactions into chorismate, the branching point metabolite for the biosynthesis of the aromatic amino acids tryptophan and phenylalanine/tyrosine (Figure 3). Most of the enzyme candidates showed a moderate and relatively stable expression level across the complete petal development. A slight increase in transcript levels (up to twofold upregulation) occurred from DS 1 to DS 3/4, followed by a decrease in DS 5. Exceptions existed, for example, in the cases of chorismate mutase and arogenate/prephenate dehydratase, where the expression was highest in the first two DSs.

Additionally, our ultra-performance liquid chromatography–high resolution tandem mass spectrometry (UPLC-HRMS/MS) data provided information on the availability of some key intermediates in the petals (Table S2). 3-Dehydroshikimate, chorismate, tryptophan, phenylalanine, and tyrosine were highly abundant at DS 1, decreased towards DS 3, and increased again towards DS 5. In contrast, anthranilate levels continually increased during petal development.

Phenylalanine and tyrosine serve as precursors of *p*-coumaric acid, which is converted to naringenin chalcone via polyketide biosynthesis. Naringenin chalcone is further transformed into naringenin and dihydrokaempferol, which finally leads to flavonols and anthocyanins. The enzyme candidates of phenylpropanoid/flavonoid biosynthesis in *P. nudicaule* were, on average, expressed two to four times higher than the enzyme candidates of the aromatic amino acid biosynthesis (Figure 4). Their levels increased in most cases towards DS 3 or 4 and strongly decreased in DS 5. Additionally, some candidates, for example dihydroflavonol 4-reductase, anthocyanidin synthase and flavonol synthase, showed stable expression levels during petal development. Similarly, acetyl-CoA carboxylase, an important enzyme of polyketide biosynthesis, was uniformly expressed.

Of the intermediates, only cinnamic acid was identified at a high level in DS 4 and 5. Of the products of flavonoid biosynthesis, the flavonol and anthocyanin pigments, five differently substituted kaempferol glycosides, one gossypetin glycoside, and two pelargonidin glycosides were identified (for structures see Figures S1 and S2). Kaempferol 3-*O*-β-d-glucoside (kaempferol 3-glc), kaempferol 3-*O*-β-d-sophoroside (kaempferol 3-soph), and kaempferol 3-*O*-β-d-[(6-*O*-malonyl)sophoroside]-7-*O*-β-d-glucoside (kaempferol 3-malsoph-7-glc) showed relatively low, stable levels over the whole petal development with maxima in DS 1 and 4 or 5. Kaempferol 3-malsoph showed a similar profile but with a tenfold higher level and a 40% decrease in DS 5. In contrast, kaempferol 3-malsoph-7-malglc, which was the main compound in DS 5, stayed at its maximum in the last two DSs. This suggests that this doubly malonylated kaempferol glycoside is the main product of flavonol biosynthesis. The less substituted derivatives, which are relatively low in abundance and/or decreasing towards DS 5, could serve as its precursors. In addition to kaempferol glycosides, gossypetin 7-*O*-β-d-glucoside (gossypitrin), a flavonol which is only present in the basal petal part [28], was identified in high amounts in DS 4 and 5. The content of pelargonidin glycosides was increasing until DS 4 and almost zero in DS 5 because of the consumption by nudicaulin production, which started in DS 4 and was highest in DS 5. Among the anthocyanins, pelargonidin 3-*O*-β-d-[(6-*O*-malonyl)sophoroside]-7-*O*-β-d-[(6-*O*-malonyl)glucoside] (pelargonidin 3-malsoph-7-malglc) was the main compound. Such a glycosidic substitution pattern was also found for the major nudicaulins VII and VIII, the (3*S*,11*R*)- and (3*R*,11*S*)-diastereomeric pairs of nudicaulin 11-*O*-β-d-[(6-*O*-malonyl)sophoroside]-7-*O*-β-d-[(6-*O*-malonyl)glucoside], respectively (Figure S3). This suggests the fusion of fully glycosylated and malonylated anthocyanin with indole, and renders glycosylation and malonylation after fusion less likely [27,44].

Generally, the metabolites of aromatic amino acid biosynthesis serve not only as precursors of nudicaulins, but also as precursors of proteins, as well as different specialised metabolites, e.g., flavonoids. Therefore, they are produced during the whole petal development. Accordingly, the corresponding amino acid biosynthesis candidate genes show a relatively stable expression throughout all DSs. In contrast, the identified early intermediates showed more variation, with higher levels at the beginning and end of petal development compared with DS 2–4. This low content in DS 2–4 correlates with high transcript levels of genes in the flavonoid pathway and an increasing pigment production, which consumes a portion of the upstream metabolites. In DS 5, shortly before anthesis, when pigment production is nearly completed, transcript levels of the flavonoid pathway, and to a smaller extent also of the aromatic amino acid/phenylpropanoid biosynthesis, are downregulated.

### 2.3. Biosynthesis of Free Indole

**Figure 5:**
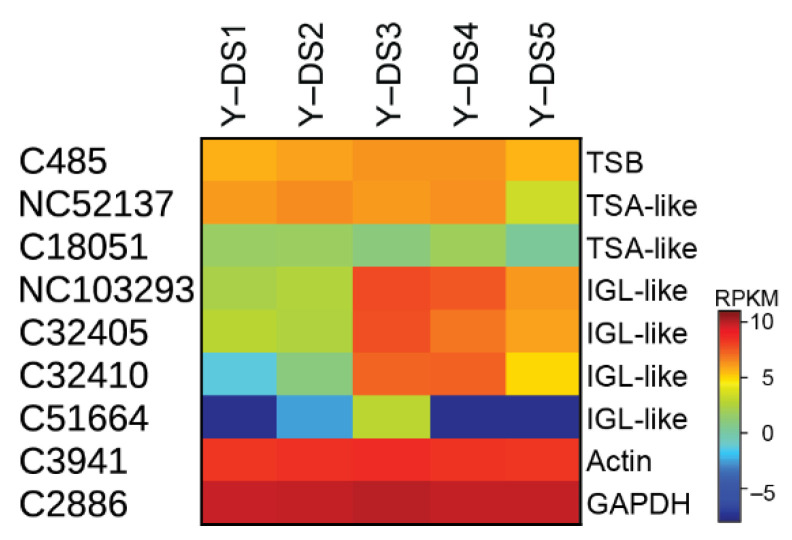
Indole-producing enzyme candidates in five DSs of the petals of yellow *P. nudicaule* flowers. The heat map shows the relative expression levels of candidate genes encoding tryptophan synthase β (TSB) and α (TSA), and indole-3-glycerol-phosphate lyase (IGL). The housekeeping genes GAPDH and actin are used for normalisation, and are shown to confirm the uniform expression of these control genes across samples. The map is based on log2-transformed RPKM values (blue represents weakly expressed genes, and red represents strongly expressed genes).

One important step in nudicaulin biosynthesis is the production of free indole, which, together with the pelargonidin glycosides, is directly incorporated into the nudicaulin scaffold. Typically, indole is formed from indole-3-glycerol phosphate by tryptophan synthase α (TSA), and subsequently, without release, channelled to tryptophan synthase β (TSB) to generate tryptophan. For the production of free indole, enzymes homologous to TSA have been found in several species, e.g., *Zea mays* [38,46], *Arabidopsis thaliana* [47], *Oryza sativa* [39], and *Consolida orientalis* [40]. These enzymes are called indole-3-glycerol-phosphate lyase (IGL) or indole synthase, and the free indole serves as a volatile or is further converted into specialised metabolites, e.g., benzoxazinoids. In yellow *P. nudicaule* petals, indole is required for tryptophan formation, nudicaulin biosynthesis, and as a volatile [27,48]. The transcriptome analysis revealed one candidate for TSB showing a relatively stable expression throughout flower development (C485, Figure 5), and two contigs homologous to TSA with a similar expression profile, which could be candidates for the α unit of tryptophan synthase (NC52137, C18051). Three other candidates homologous to TSA (NC103293, C32405, C32410) displayed rather different expression profiles, with a maximum in DS 3 and 4, similar to the profiles of the flavonoid biosynthesis candidates. These candidate enzymes could therefore catalyse the production of free indole. One other candidate (C51664) showed very low expression except in DS 3 (Figure 5).

These initial indications for two different groups of enzymes are supported by a phylogenetic analysis of TSA and IGL sequences from *P. nudicaule* and homologous sequences from other species (Figure S4). *P. nudicaule* TSA candidates cluster with protein sequences from *C. orientalis* and *A. thaliana* which were characterised to encode TSA enzymes. Likewise, *P. nudicaule* IGL candidates form a separate subcluster together with two sequences from *Papaver somniferum*, the opium poppy, which are not functionally characterised. Due to the incompleteness of several of the *P. nudicaule* sequences, gene tree associations can only be a first hint, and elucidation of the full coding sequences would be a worthwhile future task.

**Figure 6:**
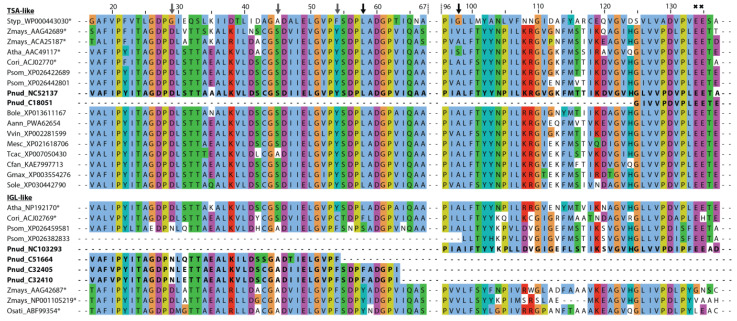
Partial sequence alignment of TSA- and IGL-like proteins of *P. nudicaule* and other species. The sequence of the *S. typhimurium* TSA is used as reference. Sequences with known function are marked with an asterisk. Potential active sites are marked with an arrow (black arrow—known sites, grey arrow—new sites). Amino acids are coloured using the Clustal X colour scheme in Jalview. Species abbreviations are: Styp—*Salmonella typhimurium*, Zmays—*Zea mays*, Atha—*Arabidopsis thaliana*, Cori—*Consolida orientalis*, Psom—*Papaver somniferum*, Pnud—*Papaver nudicaule*, Bole—*Brassica oleracea* var. *oleracea*, Aann—*Artemisia annua*, Vvin—*Vitis vinifera*, Mesc—*Manihot esculenta*, Tcac—*Theobroma cacao*, Cfan—*Carpinus fangiana*, Gmax—*Glycine max*, Sole—*Syzygium oleosum*, Osati—*Oryza sativa*. For full sequence alignment see Figure S5. Sequences of *P. nudicaule* are shown in bold.

Structure–function predictions could additionally support the presence of different indole-producing enzymes in *P. nudicaule*. In the literature, several active sites characteristic for TSA- and IGL-like enzymes have been proposed. Frey et al. identified a substitution of a conserved aliphatic leucine in the TSA sequence (position 58 of *Salmonella typhimurium* TSA) with a tyrosine in the two monomeric indole-releasing enzymes in maize, Bx1 and IGL [49]. In *P. nudicaule*, the TSA-like candidate NC52137 has a leucine at this position, whereas IGL-like candidates C32405 and C32410 have a phenylalanine instead (Figure 6). The same phenylalanine substitution can be found in the IGL of *C. orientalis*. Other deviations of maize enzymes Bx1 and IGL from the TSA consensus (e.g., substitution of two glutamic acid residues at position 134 and 135 of *S. typhimurium* TSA by valine and alanine in BX1, and by glycine and asparagine in IGL [49], marked with crosses in Figure 6) were not present in the *P. nudicaule* sequences.

Schullehner et al. proposed that the amino acid equivalent to position 98 in *S. typhimurium* possibly decides on the enzyme’s capability to produce free indole [40]. A serine was characteristic for the low activity of monomeric TSA, and valine or leucine for its high activity. An alanine at this position probably allows two conformations at this site, resulting in medium activity which is dependent on additional factors. In *P. nudicaule*, one TSA- and one IGL-like sequence (NC52137, NC103293) both possess an alanine at position 98, making them an interesting subject for further studies to identify the structural determinants of TSA-like enzymes. Due to the incompleteness of the respective mRNA sequences, the critical amino acid positions were missing in some *P. nudicaule* candidates. Additionally, positions 29, 45, and 54 show deviations from the TSA consensus in the IGL-like protein candidates of *P. nudicaule* and some of the other IGL sequences. Thus, these amino acid positions could be promising starting points for the investigation of the structure–function relationships of indole producing enzymes.

In summary, expression profiles, phylogenetic analysis, and structure–function associations of indole-producing enzyme candidates in *P. nudicaule* point towards the presence of at least one TSA-like and one IGL-like enzyme to provide indole for tryptophan and nudicaulin biosynthesis, respectively. The complete protein and gene sequences remain to be identified.

### 2.4. Concentration Dependency and Effect of the Glycosylation Pattern on the Final Step of Nudicaulin Biosynthesis

**Figure 7:**
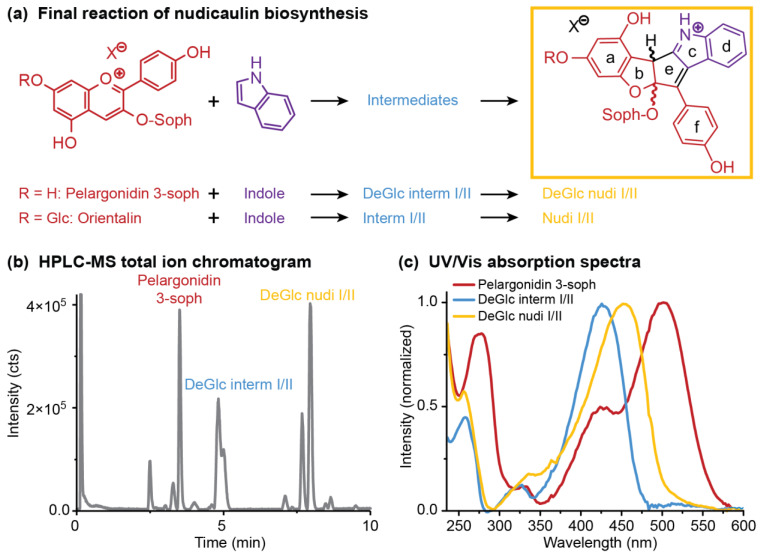
Final reaction of nudicaulin biosynthesis in vitro—pelargonidin glycosides and indole temporarily form diastereomeric intermediates (DeGlc interm I/II: 7-deglycosylated intermediates I/II; interm I/II: intermediates I/II), resulting, finally, in diastereomeric nudicaulins (DeGlc nudi I/II: 7-deglucosylated nudicaulins I/II; nudi I/II: nudicaulin I/II). (**a**) Chemical structures of educts and resulting nudicaulin derivatives (-soph: sophorose unit). (**b**) high-performance liquid chromatography–mass spectrometry (HPLC-MS) total ion chromatogram of the fusion of pelargonidin 3-soph and indole (1:1, 4 h after start of reaction, c_P_ = 10 mg mL^−1^), and (**c**) Ultraviolet/visible (UV/Vis) absorption spectra of the involved compounds. Chromatogram and spectra for the conversion of orientalin resembled the ones shown for pelargonidin 3-soph.

The last step of the nudicaulin biosynthesis seems to be, in contrast to the preceding steps, a spontaneous chemical reaction between pelargonidin glycosides and indole [27] (Figure 7a). This reaction takes place in the vacuoles of *P. nudicaule* petals under strongly acidic conditions [36]. Different ratios of nudicaulins observed in yellow and orange flowers [28] and highly different yields obtained by chemical synthesis of nudicaulin derivatives [36] and nudicaulin I and II [45] prompted us to investigate the dependence of the reaction on the concentration and the ratio of the educts. To mimic the educt present in the plant, a pelargonidin glycoside was required, which had to be extracted from the flowers. As red *P. nudicaule* flowers are known to contain these compounds as major pigments [31,50] and yellow flowers contain only small amounts during development, we chose the red cultivar ‘Matador’ to obtain the anthocyanin educt. The main compound after hydrolytic removal of malonyl substituents was isolated and identified as pelargonidin 3-*O*-β-d-sophoroside (pelargonidin 3-soph). Nuclear magnetic resonance (NMR) and high-performance liquid chromatography–mass spectrometry (HPLC-MS) data (Figures S6–S10, Table S3) matched data reported for this compound [51].

**Figure 8:**
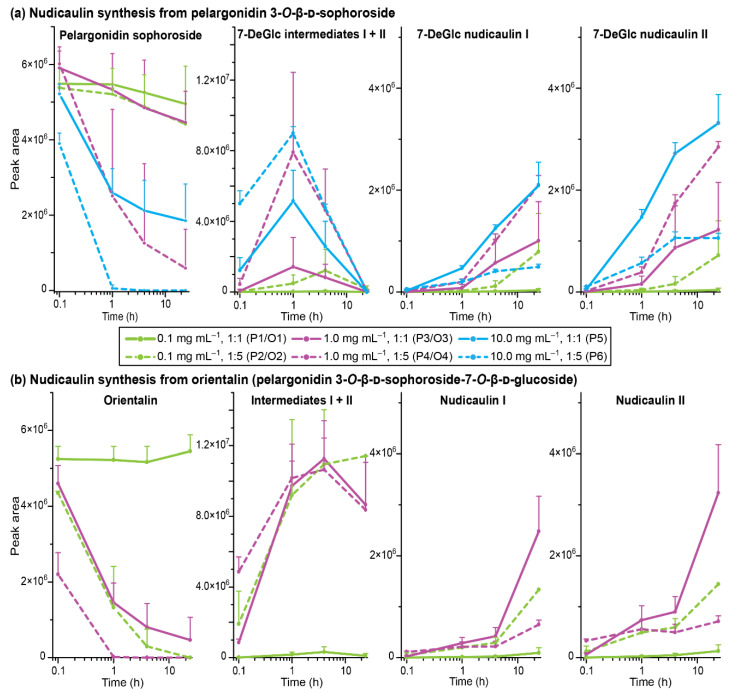
Nudicaulin synthesis (in water/1% HCOOH [*v*/*v*])—peak areas of pelargonidin glycoside, intermediates, and nudicaulins (with standard deviation), monitored by HPLC-MS (the two intermediate diastereomers are combined in one graph because the peaks were not fully separated). (**a**) Results for pelargonidin 3-soph, treatments P1–6; 7-DeGlc nudicaulin I + II: 7-deglucosylated nudicaulin I + II. (**b**) Results for orientalin, treatments O1–4.

Three different starting concentrations of pelargonidin 3-soph (0.1, 1.0, 10.0 mg mL^−1^) were used to investigate the reaction with indole. The maximum concentration was chosen based on the concentration of nudicaulins in yellow flowers of around 14 mg mL^−1^ [52]. Indole was used either in the same or fivefold molar amount, because excess of indole was also used in a previous synthetic approach [36], and the compound is continuously produced in yellow flowers and the excess emitted after anthesis [48]. The nudicaulin production was monitored over time by HPLC-MS measurements after 0.1, 1, 4, and 24 h. During this time period, the initial concentration of pelargonidin 3-soph in the reaction mixture decreased, and diastereomeric intermediates were produced and finally transformed to diastereomeric nudicaulin sophorosides, the 7-deglucosylated nudicaulins I and II (7-DeGlc nudicaulins I/II, Figure 7b, Figure 8a). The corresponding 7-deglucosylated intermediates I and II (7-DeGlc intermediates I/II) showed a mass-to-charge ratio (*m*/*z*) of 712.2248 u (calculated molecular mass *m*/*z* 712.2236 for [M+H]^+^ C_35_H_38_NO_15_^+^) (Figure S11). This is a mass difference of +2 Da in comparison to the 7-DeGlc nudicaulins I/II (Figure S12, Table S4), and points to two additional H atoms in these compounds. The ultraviolet/visible (UV/Vis) absorption spectrum of 7-DeGlc intermediates I/II resembles the one of the 7-DeGlc nudicaulins I/II, but the maximum shows a hypsochromic shift from 452 to 426 nm (Figure 7c).

For most treatments, the reaction yield increased with an increase in initial pelargonidin 3-soph concentration and with the excess of indole (Figure 8a). A concentration of 0.1 mg mL^−1^ with one equivalent of indole (treatment P1) showed only very low turnovers, and an increase of either concentration to 1.0 mg mL^−1^ (treatment P3) or of indole equivalents to five (treatment P2) increased the 7-DeGlc nudicaulin yield 20- to 30-fold. Nevertheless, 70 to 90% of the educt were still present after 24 h in these cases. Distinctly better results were achieved for treatments P4–6. Two of them (treatments P4, P5) showed similar reaction kinetics. After 1 h, more than 50% of the educt was already consumed, ending up with a 70 to 90% turnover after 24 h. In total, the highest amounts of 7-DeGlc nudicaulins were produced in these cases (treatments P4, P5). Compared to that, the highest concentration of pelargonidin 3-soph and indole in excess (treatment P6) gave only moderate yields of 7-DeGlc nudicaulins, although after 1 h the educt was already no longer detected and the highest amounts of intermediates were formed (Figure 8a). The high indole content in the solution probably led to side reactions that reduced the 7-DeGlc nudicaulin production and resulted in additional signals in the chromatogram at 9.9, 10.2, and 14.2 min (Figure S13). The two 7-DeGlc nudicaulin diastereomers seemed to differ in their reaction kinetics. In nearly all treatments, one of the diastereomers was formed in slight excess (enlarged peak areas), both for the maximum concentration point of the intermediates as well as for the final product, indicating scalemic mixtures of the diastereomers.

In *P. nudicaule* petals, nudicaulins are glycosylated at positions 7 and 11, equivalent to positions 3 and 7 in the pelargonidin educt. To investigate whether the glycosylation has an impact on the reaction kinetics, we executed an experiment using orientalin (pelargonidin 3-*O*-β-d-sophoroside-7-*O*-β-d-glucoside) instead of pelargonidin 3-soph as an educt (same molar concentration was used). Orientalin is the native precursor of nudicaulin I and II (for HRMS spectra see Figures S14–S16, Table S4). Comparison of the yields obtained with pelargonidin 3-soph and orientalin indeed pointed towards a dependence of the reaction on the glycosylation pattern (Figure 8). While the orientalin treatment O1 (c = 0.1 mg mL^−1^; ratio 1:1) resembled the results of treatment P1 and showed only a negligible turnover, O2–O4 showed differences compared to the corresponding treatments, P2–P4 with pelargonidin 3-soph. In the case of treatments O2 (0.1 mg mL^−1^; ratio 1:5) and O3 (1 mg mL^−1^; ratio 1:1) the concentration of the precursor decreased much faster—orientalin was reduced to one third after 1 h and nearly consumed after 24 h. In line with this, the level of the intermediates after 1 h was substantially higher than in treatments P2 and P3. For orientalin, the conversion of the intermediates to nudicaulins took longer than in the case of pelargonidin 3-soph—disappearance of intermediates and formation of nudicaulins accelerated after four hours, instead of after one hour. After 24 h, relatively high amounts of intermediates were still present, and the amount of nudicaulins was higher than for the less glycosylated educt originating from pelargonidin 3-soph. Treatment O4 (1 mg mL^−1^; ratio 1:5) showed a very similar course compared to O2–O3, but resulted in lower yields of nudicaulins after 24 h (Figure 8b). As assumed already for treatment P6, this might be due to the formation of side products with the fivefold indole excess.

In conclusion, the conversion of pelargonidin glycosides and indole to nudicaulins depends not only on the pH value [36], but also on the concentration, ratio of the educts, and glycosylation pattern of the pelargonidin glycoside. This might explain why nudicaulins in *P. nudicaule* occurred in DS 4 for the first time in considerable amounts, although both precursors were already present since early petal development ([27]; this publication). The reaction conditions in the petal vacuoles are probably only optimal from DS 4 onward. The fact that petals of DS 2 and 3 have a red colour, which is likely due to the pelargonidin glycosides present in a strongly acidic environment in their flavylium cation form, indicates that the acidic pH value could be already reached before DS 4. According to our data, the concentration of the educts is increasing during the flower development ([27]; this publication), probably reaching a threshold in DS 4 which is sufficient to start the nudicaulin biosynthesis in a detectable extent. Alternatively, expression of an unknown enzyme conducting the nudicaulin production only from DS 4 onward would explain why only traces of nudicaulins were detected at early DS 1–3. Furthermore, the fact that nudicaulins and their permethylated derivatives have been chemically synthesised using biomimetic conditions [36,45], as well as that the intermediates always occur as diastereomeric pairs, argues against direct enzymatic catalysis, unless a kind of enzymatic function limited to facilitate the right spatial positioning of the educts to each other might be considered.

The glycosylation pattern of the pelargonidin precursor also influences the kinetics of the reaction. As in each treatment, a pair of diastereomeric intermediates was observed (Figure 7); the conversion of intermediates to nudicaulins always seems to be the rate limiting step of the reaction cascade. However, the life-time of the intermediates was longer in the case of orientalin (treatments O2–O4) than in the case of pelargonidin 3-soph (P2–P6), where the intermediates were fully consumed after 24 h.

Our data may also explain why nudicaulins are rare in nature (or are below the detection limits) despite their precursors, anthocyanins are widespread flower pigments and indole is a common plant metabolite as well. A very acidic vacuolar pH value required for the formation of nudicaulins seems to be only one of the reasons for missing nudicaulins in most flowers. The concentration and ratio of the educts, as well as the glycosylation pattern of the pelargonidin glycosides may not only determine the reaction rate, yield, and ratio of nudicaulin diastereomers, but may be considered as criteria defining whether or not nudicaulins are formed in a certain tissue. The availability of free indole, produced and released by a special enzyme, might be an additional criterion.

It can be speculated that the excellent yields obtained by the chemical synthesis of nudicaulins I and II [45] was positively affected by using orientalin, the educt possessing the potentially optimal glycosylation pattern, conformation, and electronic properties to react with indole. Different from the synthesis of native nudicaulins, the synthesis of nudicaulin aglycon derivatives [36] applied permethylated anthocyanidin analogs with potentially suboptimal conformational and electronic properties, which may be one reason for the relatively low product yield. Another reason may be the different starting concentrations used of the pelargonidin precursor (Dudek et al. [36]: ≈ 3 × 10^−7^ mol mL^−1^, Devlin and Sperry [45]: 1.4 × 10^−5^ mol mL^−1^). The present results clearly state that a higher concentration of the pelargonidin educt would enhance the reaction yield.

**Figure 9:**
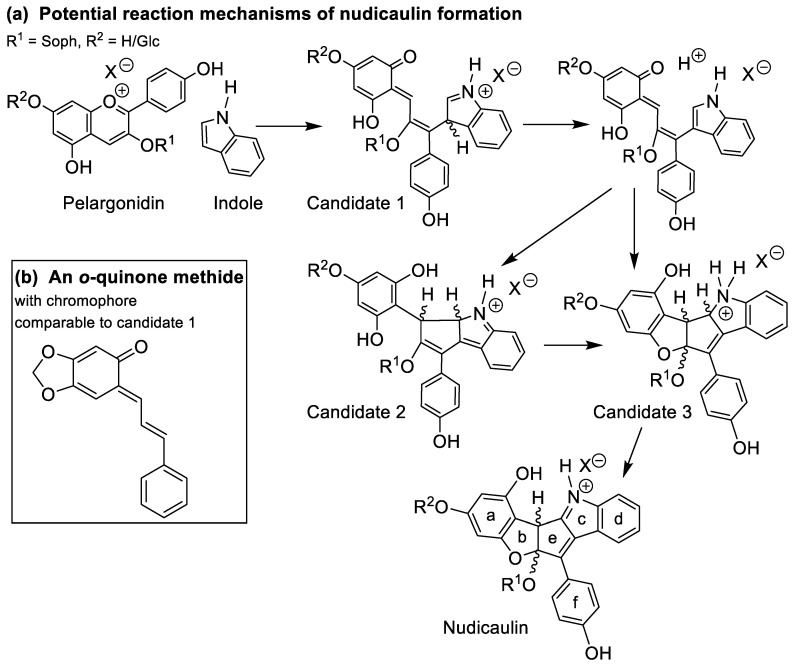
(**a**) Potential reaction mechanisms of nudicaulin formation adapted from [27] and [45]. Candidates 1–3 for the intermediate structures have a common calculated molecular mass of *m*/*z* 712.2236 [M + H]^+^ for C_35_H_38_NO_15_^+^ (R^2^ = H) and *m*/*z* 874.2764 [M + H]^+^ for C_41_H_48_NO_20_^+^ (R^2^ = β-d-glucose unit). According to our data, candidate 3 is the most likely intermediate (for details see text, Figure 7b,c and Table S4). (**b**) Structure of 6-cinnamylidene-3,4-methylenedioxy-cyclohexa-2,4-dienone, an *o*-quinone methide with a chromophore (λ_max_ = 454 nm [53]) comparable to candidate 1 in panel a.

In order to substantiate the mechanism of the nudicaulin biosynthesis, the structure of the intermediates remains to be assigned. The measured molecular mass *m*/*z* 712 (Figure S11) points to two additional H atoms compared to nudicaulins, and suggests that the intermediates are oxidatively transformed into the final nudicaulin structure. In addition to the molecular mass, the detection of two isomers, likely being diastereomers, by HPLC (Figure 7b) and the UV/Vis data are valuable evidences to hypothesise the structure of the intermediate. The UV/Vis absorption spectrum (Figure 7c) of the compounds is similar to the spectrum of the nudicaulins, but shows a hypsochromic shift of the maximum to 420 nm resulting in a pale yellow colour. This indicates a less conjugated chromophore compared to the nudicaulins. Possible structures with the corresponding mass were proposed by Warskulat et al. [27] (Figure 9a). Candidate 1 has a chromophoric system comparable to *o*-quinone methides, e.g., 6-cinnamylidene-3,4-methylenedioxy-cyclohexa-2,4-dienone (Figure 9b) which has an orange colour, λ_max_ = 454 nm [53]. Thus, candidate 1 is expected to absorb at longer wavelengths than observed for the intermediates. For candidate 2, no absorption maximum in the visible spectral range can be expected because of the limited extension of the chromophore. Another possible intermediate structure (candidate 3) was proposed by Devlin and Sperry [45]. This structure closely resembles the one of the nudicaulins, just lacking one double bond in the chromophore. Thus, candidate 3 (Figure 9a) is, judging from the size of the chromophore, the most reasonable structure for the intermediate.

### 2.5. Comparative Metabolome and Transcriptome Analysis of Differently Coloured P. nudicaule Cultivars

In *P. nudicaule*, not only flowers from yellow, but also from orange cultivars contain nudicaulins [31]. The petal development of orange flowers resembles that of the yellow flowers as the petals inside the buds also show a colour shift from white over pale red and red to orange. Shortly after reaching the orange colour, flowers start blooming. The orange petals still contain pelargonidin glycosides, but lack free indole [48]. The white *P. nudicaule* cultivar contains kaempferol derivatives and indole but lacks pelargonidin derivatives whereas the red cultivar contains both kaempferol and pelargonidin derivatives as well as low levels of indole during the petal development [28,48]. Up to now, the pigments have only partly been identified for the petals of the orange, red, and white cultivar during development and flowering. The fact that the red cultivar lacks nudicaulins although both precursors are present during the development, and that the orange cultivar produces nudicaulins only to a certain extent suggest discrete regulation of pigment biosynthesis in differently coloured petals.

**Figure 10:**
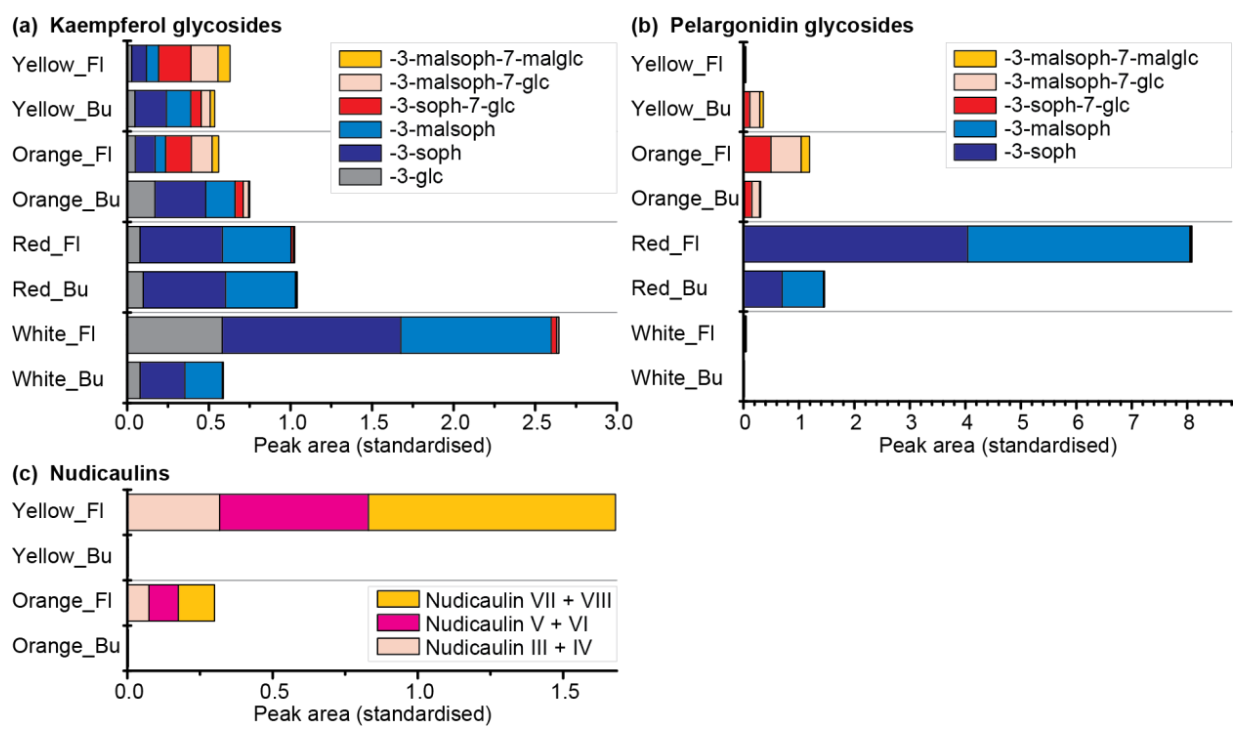
Flavonoids and nudicaulins in buds (Bu, DS 3) and flowers (Fl) of the yellow, orange, red, and white *P. nudicaule* cultivars (mean values, see Table S5). (**a**) Kaempferol glycosides with different substituents: 3-*O*-β-d-sophorosyl unit—3-soph, 3/7-*O*-β-d-glucosyl unit—3/7-glc, 6-*O*-malonyl unit—mal. (**b**) Pelargonidin glycosides with different substituents. (**c**) Nudicaulins: nudicaulins III + IV are bearing a 11-*O*-β-d-[(6-*O*-malonyl)sophorosyl] and a 7-*O*-β-d-glucosyl unit; nudicaulins V + VI are bearing a 11-*O*-β-d-sophorosyl and a 7-*O*-β-d-[(6-*O*-malonyl)glucosyl] unit; nudicaulins VII + VIII are bearing a 11-*O*-β-d-[(6-*O*-malonyl)sophorosyl] and a 7-*O*-β-d-[(6-*O*-malonyl)glucosyl] unit. For structures of the pigments see Figures S1–S3.

Therefore, a comparative metabolomics approach of DS 3 and blooming flowers of the yellow, orange, red, and white cultivars was conducted (Table S5). In total, six kaempferol and five pelargonidin glycosides were identified (Figure 10a,b; for structures see Figures S1 and S2). Of those, 3-glycosylated derivatives dominated in the white and red cultivars, whereas 3,7-diglycosylated derivatives dominated in the orange and yellow cultivars. Kaempferol glycosides were present in all four cultivars, with the highest concentration in white flowers. Kaempferol 3-soph and kaempferol 3-malsoph were the main compounds in buds as well as flowers of white and red *P. nudicaule*. Additionally, they were prevalent in the buds of orange and yellow *P. nudicaule*, but their amount decreased in blooming flowers, where kaempferol 3-soph-7-glc and a mono- and dimalonylated derivative dominated (Figure 10a). The levels of pelargonidin glycosides were highest in the flowers of the red cultivar, which contained pelargonidin 3-soph and pelargonidin 3-malsoph. In contrast, buds and flowers of the orange cultivar as well as buds of the yellow cultivar contained orientalin and malonylated forms thereof. In the flowers of the yellow and in flowers and buds of the white cultivar, only traces of pelargonidin glycosides were found (Figure 10b). Nudicaulins were, as expected, only present in flowers of the yellow and orange cultivar in considerable amounts, and absent or present in traces in the other samples (Figure 10c). The most abundant compounds were the diastereomers nudicaulin VII and VIII (substituted with a 11-malsoph and a 7-malglc), which is in accordance with Schliemann et al. [54]. All natural nudicaulins identified so far bear a sophorose as well as a glucose moiety [31,54], which reflects the glycosylation pattern of the direct precursors, the (malonylated) pelargonidin 3-soph-7-glcs (for structures see Figures S2 and S3). The present metabolome analysis showed that these diglycosylated precursors are limited to nudicaulin-containing cultivars of *P. nudicaule*, which suggests that the glycosylation at position 7 of the pelargonidin may favour the nudicaulin production. Consequently, the red cultivar, which contained only traces of diglycosylated pelargonidin derivatives, also showed only traces of nudicaulins. The monoglycosylated pelargonidin derivatives were not converted to nudicaulins although indole is present during petal development. In case of the white cultivar, the general absence of pelargonidin glycosides accounts for the lack of nudicaulins, whereas in case of the orange cultivar, a low amount of indole is the limiting factor for nudicaulin production.

At the transcript level, a comparison of the yellow cultivar to the second nudicaulin-producing, orange cultivar, and to the white cultivar, which lacks both anthocyanins and nudicaulins, was performed. For orange and white cultivars five DSs were also defined according to colour and day of development; DS 5 represented the blooming flower. A transcriptome-wide cluster analysis clearly separated the white cultivar from the other cultivars. Likewise, DS 1–4 of the yellow and the orange cultivar each occupied one subcluster. DS 5 of those two cultivars showed overall higher gene expression profile similarity to each other than to the DS of the same cultivar (Figure S17).

Candidate genes belonging to the nudicaulin and flavonoid biosynthesis were similarly expressed in all three cultivars (Table S1). The expression of only a few candidates deviated by more than four times from the expression of the yellow cultivar. For example, shikimate kinase (C2405) and arogenate/prephenate dehydrogenase (C5470) were expressed more highly in the yellow cultivar than in the orange and white ones. For the phenylpropanoid and flavonoid biosynthesis, the yellow and orange cultivars showed substantially higher gene expression than the white cultivar for about half of the candidates and DS. As all three cultivars produce flavonols, similar expression levels of genes encoding enzymes for the necessary biosynthetic steps is reasonable. The additional presence of anthocyanins in the yellow and orange cultivars in comparison to the white one might be the reason for the higher expression of the phenylpropanoid/flavonoid biosynthesis in those cultivars. Nevertheless, anthocyanin gene expression is not fully downregulated in white flowers, which might explain why some white flowers showed a pale red colour—sometimes during development, sometimes during flowering. In the case of the orange blooming flowers, the similar gene expression patterns to yellow blooming flowers reflect the similar pigment composition during development. Orange flowers do not convert all pelargonidin glycosides into nudicaulins, probably due to a lower indole production. Interestingly, the indole biosynthesis genes of the orange cultivar are not downregulated in comparison to the yellow one. Only for three IGL candidates (C32405, C32410, and NC103293) expression in DS 5 was much lower in the orange than in the yellow cultivar, suggesting that the synthesis of free indole might stop earlier in the orange cultivar than in the yellow one. According to these data, expression of biosynthetic genes is another factor influencing the nudicaulin production in addition to pH value, concentration, ratio of the educts, and glycosylation pattern of the pelargonidin glycosides.

## 3. Materials and Methods

### 3.1. Plant Material

*Papaver nudicaule* L. plants were raised from seeds of ‘Summer Breeze Yellow’, ‘Summer Breeze Orange’, ‘Wonderland White’, and ‘Matador Red’ (Jelitto Staudensamen, Schwarmstedt, Germany) in soil (clay substratum and TS1 substratum, Klasmann-Deilmann, Geeste, Germany) in the glasshouse facilities of the Max Planck Institute for Chemical Ecology, Jena, Germany. The glasshouse chamber was equipped with Phillips Sun-T Agro 400 Na lights to support an illumination period of 14 h. Temperatures ranged from 19–21 °C in the night and from 21–23 °C during the day with a relative air humidity of 50–60%. Plants were irrigated for 6 min every day.

### 3.2. Definition of Developmental Stages of Yellow P. nudicaule Petals

Buds and freshly opened flowers were harvested and stored on ice (56 yellow cultivar samples). Afterwards, the petals were picked and the basal areas were removed. The samples were weighted and a solvent equivalent of H_2_O and MeOH (7:3 [*v*/*v*]) was added to achieve a uniform concentration of 1 mg petal material per 10 µL solvent. After grinding in a homogeniser (MINILYS personal homogenizer, 60 s, full speed, Bertin Technologies S.A.S., Montigny-le-Bretonneux, France), the petals were extracted in ultrasonic bath for 15 min with ice cooling, followed by centrifugation (15 min; 4 °C; 13,400 rpm). The supernatants were used for the HPLC-PDA analysis applying kaempferol (Sigma-Aldrich, Steinheim, Germany) as an internal standard with the concentration of 0.05 µg mL^−1^. The analytical HPLC-PDA system consisted of an Agilent series HP1100 (degasser G1322A, binary pump G1312A, autosampler G1313A, and photodiode array detector G1315B, 200–700 nm, Waldbronn, Germany) equipped with an EC250/4 Nucleodur C18 HTec column from Macherey-Nagel (5 µm particle size, injection volume 20 µL, Düren, Germany). The method included a 38 min gradient from 0 to 76% of MeOH in acidified H_2_O (0.1% TFA [*v*/*v*]), changing to 100% MeOH in 2 min with a subsequent washing step (100% MeOH, 5 min) and equilibration to starting conditions (5 min gradient to 0% MeOH, 5 min 0% MeOH). The flow rate was 1 mL min^–1^ and 211, 254, 281, 351 and 460 nm were used as detection wavelengths.

The data set for multivariate-statistical examination was obtained from HPLC-PDA of raw extracts. Most peaks were detected at a wavelength of 254 nm, therefore this detection wavelength was chosen for further analysis. For each sample all occurring peaks detected by the software Agilent LC ChemStation B.04.01 were summarised including retention time and peak area (t_r_ ≥ 6 min, peak area > 50). Peaks with similar retention times in different samples were compared by their UV spectra and combined if representing the same compound. Zero values were replaced by a value of 10 (see Table S6). The resulting data set was standardised by subtraction of the total mean value from each measurement value and division by the standard deviation and used for cluster analysis. Hierarchical agglomerative clustering was performed by SciPy version 1.4.1 [55] using unweighted average linkage clustering as fusion algorithm along with Euclidean distance, and the dendrogram was visualised with Matplotlib version 3.2.0 [56].

### 3.3. Transcriptomics

#### 3.3.1. RNA-Seq and Differential Gene Expression Analysis

Transcriptome analysis was performed on white, yellow, and orange *P. nudicaule* flowers. After harvesting, one sample for each DS was chosen according to colour and day of development. In the case of the yellow cultivar, DS were separated into white, pale red, red, orange, and yellow buds following the classification described in RESULTS (definition of stages in the development of yellow *P. nudicaule* petals). The colour change during development of the orange cultivar resembled that of the yellow one, and buds were harvested on day 6 (white), 9 (pale pink), 11 (pale red), 13 (red), and a freshly blooming flower (orange) for the analysis. In the case of the white cultivar, which does not show a colour change, the samples were also collected on days 6, 9, 11, 13, and the blossoming day.

For RNA extraction petals were carefully picked, submerged in 450 µL lysis buffer (innuPREP RNA Mini Kit, Analytik Jena GmbH, Jena, Germany), frozen with liquid N_2_, and stored at −80 °C until usage. After thawing, samples were homogenised in a TissueLyser LT (Qiagen GmbH, Hilden, Germany) at 50 Hz for 3 min (2 steel balls, 3 mm diameter), and centrifuged (1 min; 10,000× *g*; room temperature). RNA was isolated using the innuPREP RNA Mini Kit according to the manufacturer’s instructions, while genomic DNA was removed from the supernatant via solid phase extraction. The integrity of the RNA was verified using an Agilent 2100 Bioanalyzer and an RNA 6000 Nano Kit (Agilent Technologies, Waldbronn, Germany). The quantity of RNA was determined using a Nanodrop ND-1000 spectrophotometer (PEQLAB Biotechnologie GmbH, Erlangen, Germany). All samples complied with the purity and RNA integrity requirements for transcriptome analysis.

Transcriptome sequencing was carried out by the Max Planck Genome Centre Cologne (Köln, Germany) on an Illumina HiSeq2500 Genome Analyzer platform, using paired-end (2 × 100 bp) read technology for the 15 flower samples, yielding ≈ 15 million reads for each sample. Quality control measures and de novo transcriptome assembly, combining all RNA-Seq samples, were carried out using CLC Genomics Workbench v9.1 (http://www.clcbio.com, accessed on 4 December 2014) as previously described [57]. The de novo reference transcriptome assembly of *P. nudicaule* contained 52,906 contigs with an N50 contig size of 1255 bp and a maximum contig length of 14,346 bp. The transcriptome was annotated using BLAST, Gene Ontology and InterProScan searches (accessed on 13 November 2019) implemented in BLAST2GO PRO v5.1 (http://www.blast2go.com) [58]. For BLASTx searches against the nonredundant NCBI protein database (NR database), up to 20 best NR hits per transcript were retained, with an E-value cutoff of ≤10^−3^ and a minimum match length of 15 amino acids.

To assess transcriptome completeness, we performed a BUSCO (benchmarking universal single-copy orthologs; http://busco.ezlab.org, accessed on 4 December 2014) analysis by comparing our assembled transcriptome against a set of highly conserved single-copy orthologs. This was accomplished using the BUSCO v3 pipeline [59], comparing the predicted proteins of the *P. nudicaule* transcriptome to the predefined set of 1438 Embryophyta single-copy orthologs from the OrthoDB v9.1 database. This resulted in 76.5% complete and 8.5% missing BUSCO genes for the transcriptome assembly of *P. nudicaule* flowers.

Digital gene expression analysis was carried out using CLC Genomics workbench v9.1 to remap the Illumina reads from all 15 samples onto the *P. nudicaule* reference transcripts to generate BAM mapping files, and finally by counting the sequences to estimate expression levels, using previously described parameters for read mapping and normalisation [60].

#### 3.3.2. Phylogenetic Analysis of Plant TPS/IGL-like Protein Sequences

We inferred the species-specific diversification patterns of putative TPS/IGL-like protein sequences identified in the *P. nudicaule* flower transcriptome in phylogenetic analyses. We first obtained previously characterised plant TPS/IGL sequences from Genbank and further used all predicted *P. nudicaule* TPS/IGL-like protein sequences as a query to search for homologs in the NCBI nr protein database using Blastp (E-value threshold of 10^−10^), identified the top 50 best Blast hits, and removed redundant entries. Next, we removed partial sequences with less than 50% of the typical TPS/IGL length. The corresponding protein sequences were aligned using MAFFT implemented in Geneious (v11.0.4) with FFT-NS-ix1000 algorithm and BLOSUM62 scoring matrix. The alignments were viewed in Jalview version 2.11.1.2, trimmed manually, and amino acids were coloured using the Clustal X colour scheme. Phylogenetic relationships were inferred by maximum likelihood analysis using IQ-TREE version 1.6.12 [61] with the VT+G+F substitution model. Likelihood-based support of tree nodes was generated using an approximate likelihood-ratio test (SH-aLRT), ultrafast bootstrap, and approximate Bayes’ approach implemented in IQ-TREE.

#### 3.3.3. Data Accessibility

The short read data have been deposited in the EBI short read archive (SRA, upload on 12 January 2021) with the following sample accession numbers: ERS5547893-ERS5547907. The complete study can also be accessed directly using the following URL: http://www.ebi.ac.uk/ena/data/view/PRJEB42429.

Proteins of the target biosynthetic pathways were extracted from KEGG PATHWAY database (KEGG 86.0, Kanehisa Laboratories, Kyoto, Japan), and the transcriptome dataset was screened for the respective EC numbers and protein names. From all hits, the best candidates were selected according to the longest representative sequences by inspecting the respective alignments and co-occurrence in the proteomics dataset. In cases where several contigs with the same expression profile were obtained, only one representative contig was shown in the figures.

### 3.4. Proteomics

#### 3.4.1. Protein Extraction and 2D Differential Gel Electrophoresis (2D-DIGE)

Chemicals for proteome analysis were purchased from Carl Roth GmbH & Co. KG (Karlsruhe, Germany) and Sigma-Aldrich (Steinheim, Germany) if not stated otherwise. Buds of the yellow *P. nudicaule* cultivar were harvested and stored on ice. Petals were picked, sorted according to their DS, pestled into a fine powder under cooling with liquid nitrogen, and stored at −80 °C. Phenol extraction of proteins followed the protocol by Faurobert et al. [62] using 3 g tissue instead of 1 g and the respective buffer/solvent volumes.

The proteins were resuspended in isoelectric focussing buffer (7 M urea, 2 M thiourea, 2% CHAPS, 20 mM Tris, 0.5% Pharmalytes pH 3–11) and were agitated for 1 h on a thermoshaker. The concentration of proteins was determined by Bradford assay (Coomassie Plus Protein Assay Reagent, Thermo Fisher Scientific Inc., Waltham, MA, USA). To enable a direct comparison between the different DSs, samples were subjected to 2D-differential gel electrophoresis (2D-DIGE), following the protocol of Wartenberg et al. [63]. The Refraction-2D^TM^ Labeling Kit (NH DyeAGNOSTICS GmbH, Halle, Germany) was used. For details of the 2D-DIGE see Methods S1.

#### 3.4.2. Protein Digestion and HPLC-MS Analysis

Protein bands/spots were cut from the gel matrix and tryptic digestion was carried out as reported by Shevchenko et al. [64]. For HPLC-MS analysis, the extracted tryptic peptides were reconstructed in 10 µL acidified H_2_O (1% HCOOH [*v*/*v*]). Depending on the staining intensity, 1 to 5 µL of sample was injected into the HPLC-MS/MS system. The data were acquired on a nanoAcquity UPLC system connected online to a Q-ToF Synapt HDMS mass spectrometer (Waters, Milford, MA, USA). The peptides were concentrated on a Symmetry C18 trap-column (20 × 0.18 mm, 5 µm particle size, Waters) using a mobile phase of H_2_O (0.1% HCOOH [*v*/*v*]) at a flow rate of 15 µL min^−1^ and separated on a nanoAcquity C18 column (200 mm × 75 µm ID, C18 BEH 130 material, 1.7 µm particle size, Waters) by in-line gradient elution at a flow rate of 0.35 µL min^−1^ using the following gradient: 1–40% B within 40 min, 40–80% B within 10 min, 80–95% B within 1 min, isocratic at 95% B for 2 min, and a return to 1% B (Buffers: A, H_2_O/0.1% HCOOH [*v*/*v*]; B, CH_3_CN/0.1% HCOOH [*v*/*v*]).

The eluted peptides were transferred to the nanoelectrospray source of a Synapt HDMS tandem mass spectrometer (Waters) which was operated in V-mode with a resolution power of at least 10,000 FWHM. All analyses were performed in positive ESI mode. Human Glu-fibrinopeptide B (650 fmol μL^−1^) in H_2_O/0.1% HCOOH [*v*/*v*]—CH_3_CN (1:1 [*v*/*v*]) was infused at a flow rate of 0.5 μL min^−1^ through the reference Nano-LockSpray source every 30 s to compensate for mass shifts in MS and MS/MS fragmentation mode. HPLC-MS data were collected using data-dependent acquisition. The acquisition cycle consisted of a survey scan covering the range of *m*/*z* 400–1800 Da followed by MS/MS fragmentation of the five most intense precursor ions collected at 1.5 s intervals in the range of 50–2000 *m*/*z*. Dynamic exclusion was applied to minimise multiple fragmentations for the same precursor ions.

#### 3.4.3. Data Processing and Protein Identification

Data dependent aquisition raw data were processed and searched against a subdatabase containing common contaminants (human keratins and trypsin) using ProteinLynx Global Server (PLGS) version 2.3 (Waters). The following search parameters were applied: fixed precursor ion mass tolerance of 15 ppm for survey peptide, fragment ion mass tolerance of 0.1 Da, 1 missed cleavage, fixed carbamidomethylation of cysteines, and possible oxidation of methionine. Spectra that remained unmatched by database searching were interpreted de novo to yield peptide sequences and subjected for homology-based searching using the MS BLAST programme [65] installed on a local server. MS BLAST searches were performed against a *P. nudicaule* subdatabase obtained from in silico translation of the *P. nudicaule* transcriptome and against the *plantae* database downloaded from https://www.ncbi.nlm.nih.gov/ accessed on 4 December 2014.

In parallel, pkl-files of MS/MS spectra were generated and searched against the *P. nudicaule* subdatabase combined with NCBInr database containing 53,331,289 sequences downloaded from https://www.ncbi.nlm.nih.gov/ accessed on 4 December 2014. The database searching was performed using MASCOT software version 2.3 (Matrix Science Ltd., London, UK) using the search parameters described above.

### 3.5. Metabolomics

#### 3.5.1. Sample Preparation and Metabolomics Analysis of Five Developmental Stages of Yellow *P. nudicaule* Flowers

For metabolomic measurements of the five DSs of yellow *P. nudicaule* flowers, buds were harvested, stored on ice, opened, and sorted according to their colour. The petals of five buds per sample were picked and ground under cooling with liquid N_2_. For each DS, three replicates were taken and stored at −80 °C. Afterwards, 20–40 mg of the tissue was weighed and extracted with MeOH–H_2_O 1:1 (resulting concentration c = 40 µL mg tissue^−1^) for 30 min in an ultrasonic bath under ice cooling. The solvent contained 20 µmol L^−1^ of the internal standards indole-3-propionic acid (Sigma-Aldrich, Steinheim, Germany) and [1-^13^C]L-leucine (Sigma-Aldrich). After extraction, samples were centrifuged (20 min; 4 °C; 13,200 rpm) and the supernatant was subjected to UPLC-HRMS/MS analysis.

UPLC-HRMS/MS measurements were recorded on an LTQ-Orbitrap™ XL Mass Spectrometer (Thermo Fisher Scientific, Bremen, Germany) operated in positive and negative ion mode, with chromatographic separation done on an Ultimate 3000 series RSLC (Dionex, Sunnyvale, CA, USA) system. The chromatographic settings were the same as for the DS 3 and flower analysis (see next paragraph). The ESI source parameters were as follows: A spray voltage of 5 kV, capillary temperature of 275 °C, tube lens of 110 V, and full scan range of *m*/*z* 80–1200 at 30,000 m/Δm resolving power.

#### 3.5.2. Sample Preparation and Metabolomics Analysis of Developmental Stage 3 and Flower of Four *P. nudicaule* Cultivars

Comparative metabolomic analysis of the yellow, orange, red, and white cultivars was performed with the petals of open flowers and buds of DS 3 (pale red colour, or day 12 of development in the case of white cultivar). One whole flower per sample was used of DS 3 and one petal per sample of blooming flowers (three replicates each). Flowers/buds were harvested and stored on ice. Petals were picked, weighed, and solvent (MeOH–H_2_O 1:1, containing 20 µmol L^−1^ each of internal standards indole-3-propionic acid and [1-^13^C]L-leucine) was added to reach a concentration of 40 µL mg tissue^−1^. Samples were vortexed, sonicated for 30 min in an ice bath, centrifuged (20 min; 4 °C; 13,200 rpm), and the supernatant was subjected to UPLC-HRMS/MS analysis.

UPLC-HRMS/MS measurements were recorded on a Q Exactive™ HF-X Hybrid Quadrupole-Orbitrap™ Mass Spectrometer (QE-HF-X) (Thermo Fisher Scientific, Bremen, Germany) operated in positive and negative ion mode, with chromatographic separation done on an Ultimate 3000 series RSLC (Dionex, Sunnyvale, CA, USA) system. The UPLC measurements were conducted on an Acclaim C18 column (150 × 2.1 mm, 2.2 µm particle size with 120 Å pore diameter (Dionex) with a flow rate of 200 µL min^−1^ in a binary solvent system of water (Solvent A) and acetonitrile (Solvent B), both containing 0.1% [*v*/*v*] formic acid. Extracts (5 µL) were loaded onto the column and eluted by using a gradient as follows: 0.5% B for 3.5 min; linear increase from 0.5 to 35% B within 11.5 min; 35 to 100% B within 2.5 min; 100% B for 2 min; 100 to 0.5% B within 2.5 min; equilibration at 0.5% B for 3 min. The ESI source parameters were as follows: a spray voltage of 2.5 kV, capillary temperature of 300 °C, Funnel RF of 40 V and fragmentation was done using data-dependent acquisition mode with MS1 full scan at *m*/*z* 80–1200 at 60,000 m/Δm resolving power and up to five MS/MS scans (TOP5) of the most abundant ions per duty cycle with 30,000 m/Δm resolving power and stepped normalised collision energy of 20, 30, and 40.

#### 3.5.3. Data Analysis and Candidate Metabolite Identification

Both datasets were evaluated and interpreted using Xcalibur v.3.0.63 software (Thermo Fisher Scientific, Waltham, MA, USA). A mass tolerance of ±4 ppm was used as the threshold between accurate and exact mass. The UPLC-HRMS raw spectra were first centroided by converting them to mzML format using the MS Convert feature of ProteoWizard 3.0.18324. Data processing was subsequently carried out with R Studio v1.1.463 using the Bioconductor XCMS package v 3.4.2, which contains algorithms for peak detection, peak deconvolution, peak alignment, and gap filling. The resulting peak list values were used to generate graphical plots. Peak areas were standardised by division by the peak area of the internal standard [1-^13^C]L-leucine. For heatmaps of the DSs of yellow *P. nudicaule*, mean values relative to the highest value of each compound or, in the case of kaempferol and pelargonidin glycosides and of nudicaulins, relative to the highest value of all metabolites with the same aglycon were used.

The presence of each feature of interest was confirmed in the raw data and the molecular formula estimated. This information, along with retention time, accurate mass, and MS/MS spectra were used to probe into the existing literature and databases. MS/MS spectra files were also centroided and imported into the Global Natural Products Social Molecular Networking platform (GNPS https://gnps.ucsd.edu/; 21–22 July 2019) for spectral matches and classical molecular networking. Datasets are available at GNPS:

- Pnudicaule_YFP_DV1-5_Bud_neg_xcms-GNPS https://gnps.ucsd.edu/ProteoSAFe/status.jsp?task=c3f0d47205f449b6918b323816539307
- Pnudicaule_YFP_DV1-5_Bud_pos_xcms-GNPS https://gnps.ucsd.edu/ProteoSAFe/status.jsp?task=5489ef2fdc4743d093e25cb80700c39f
- Pnudicaule_DV3_Flower-Bud_pos_xcms-GNPS https://gnps.ucsd.edu/ProteoSAFe/status.jsp?task=28eee1f5df1547dbad02cc1693741b07
- Pnudicaule_DV3_Flower-Bud_neg_xcms-GNPS https://gnps.ucsd.edu/ProteoSAFe/status.jsp?task=fc1f75e783be4946827c83bd90f9e528

MS/MS-based molecular networks were generated using the GNPS online platform [66]. An ion mass tolerance and a fragment ion mass tolerance of 0.02 Da were used. MS-clustering was on, and a spectral library search was performed using 0.02 Da for ion mass and fragment ion tolerance. A minimum cosine score of 0.7 was set for achieving spectral matching with the MS/MS spectral libraries and a minimum of six matching peaks was considered for spectral library annotations.

The dereplication process was achieved by searching for the MS/MS spectra in the GNPS libraries. This network was imported to Cytoscape version 3.6.1 (https://www.cytoscape.org) and analysed using default algorithms and other visual considerations.

### 3.6. Isolation and Identification of Pelargonidin Glycosides

Pelargonidin 3-soph was isolated from red *P. nudicaule* flowers. Therefore, the frozen petals were ground under cooling with liquid N_2_ and extracted two times for 20 min under ultrasound with an acidified 1:1 mixture of MeOH (HPLC-gradient grade, Sigma-Aldrich, Steinheim, Germany) and deionised H_2_O (ultrapure water Milli-Q, 0.1% HCOOH ([*v*/*v*], 99% for HPLC-MS, VWR International, Darmstadt, Germany)). The sample was evaporated to dryness in vacuo. Afterwards, anthocyanins were purified by solid phase extraction. The crude extract was dissolved in acidified H_2_O (1% HCOOH [*v*/*v*]) and loaded on Chromabond drug cartridges (Macherey-Nagel, Düren, Germany). Washing with H_2_O (1% HCOOH [*v*/*v*]) removed polar compounds, and washing with a mixture of MeOH–H_2_O 4:6 (0.1% HCOOH [*v*/*v*]) removed non-ionic flavonoids. Anthocyanins were eluted with acidified MeOH (1% HCl [*v*/*v*], 37%, Sigma-Aldrich) and dried under a stream of N_2_ gas.

For split-off of malonyl groups, the sample was dissolved in an aqueous NaOH solution (10% [*m*/*v*], purity ≥99%, Sigma-Aldrich, c ≈ 1 mg anthocyanin mL^−1^). A 15 min reaction time in the dark was followed by neutralisation of the solution with HCl (37%, colour change from dark yellow to red), and drying under a stream of N_2_. Demalonylation resulted in two pelargonidin glycosides. These compounds were trapped on a Chromabond C_18_ ec cartridge (Macherey-Nagel); malonic acid was removed by washing with acidified H_2_O (0.1% HCOOH [*v*/*v*]), and anthocyanins were eluted with H_2_O–MeOH 1:1 (0.1% HCOOH [*v*/*v*]). The final separation was performed by preparative HPLC on a Shimadzu Prominence HPLC system (Shimadzu, Duisburg, Germany) controlled by the Shimadzu LCSolution software, ver. 1.21, and consisting of a DGU-20A5 degasser, LC-20AT gradient pump, SIL-10AP autosampler, CTO-20A column oven, SPD-20A UV detector, FRC-10A fraction collector, and CBM-20A system controller. A VP 250/10 Nucleodur C18 HTec column (5 µm; Macherey-Nagel) was used at 25 °C with a flow rate of 3.5 mL min^−1^. The binary solvent system consisted of acidified H_2_O (0.1% HCOOH [*v*/*v*], solvent A) and acidified MeOH (0.1% HCOOH [*v*/*v*], solvent B). The method included a focussing step of 5 min with 30% solvent B, followed by a linear binary gradient from 30–70% B within 8 min, changing to 100% within 3 min with a subsequent washing step (100% B, 7 min) and equilibration to starting conditions (2 min gradient to 30% B, 5 min 30% B). The sample was dissolved in H_2_O–MeOH 6:4 (0.1% HCOOH [*v*/*v*], c ≈ 48 mg mL^−1^) and fraction collection was triggered by peak intensity using detection at 460 nm and time. The structure of the main compound, pelargonidin 3- soph, was elucidated by NMR on a Bruker Avance III HD 500 MHz spectrometer (operating at 500.13 MHz for ^1^H and 125.75 MHz for ^13^C) equipped with a 5 mm TCI cryoprobe. ^1^H NMR, ^1^H,^1^H-COSY, HSQC, and ^1^H,^13^C-HMBC spectra (standard Bruker pulse sequences as implemented in Bruker TopSpin) were measured in MeOH-*d*_4_ with 2% TFA-*d*_1_ as solvents. Chemical shifts were referenced to the residual solvent peaks at δ_H_ 3.31 and δ_C_ 49.15 for MeOH-*d*_4_ (for spectra see Figures S6–S10, Table S3). Data acquisition and processing were accomplished using TopSpin 3.5 (Bruker Biospin, Rheinstetten, Germany). Orientalin chloride was kindly provided by Prof. Jonathan Sperry, University of Auckland, New Zealand. HPLC-PDA-HRESIMS measurements of pelargonidin 3-soph and orientalin are described in the following paragraph.

### 3.7. Concentration Dependency of the Final Step of Nudicaulin Biosynthesis

For the in vitro synthesis of nudicaulins I/II and their 7-deglucosylated derivatives, stock solutions of the educts were prepared: pelargonidin 3-soph formate and orientalin chloride in H_2_O (1% HCOOH [*v*/*v*]), c_P_/c_O_ = 10 mg mL^−1^; indole in MeOH, c_I1_ = 5 mg mL^−1^, c_I2_ = 0.1 mg mL^−1^. Respective amounts of indole solution were pipetted into 0.5 mL tubes (Eppendorf, Hamburg, Germany) and the solvent was evaporated under a gentle stream of N_2_ gas. Afterwards, H_2_O (1% HCOOH [*v*/*v*]) and pelargonidin stock solution were added to reach the following concentrations: pelargonidin 3-soph formate, treatments P1/2, P3/4, P5/6 c_P_ = 0.10, 1.00, and 10.00 mg mL^−1^; orientalin chloride, treatments O1/2, O3/4 c_O_ = 0.12, 1.24 mg mL^−1^ (molar concentration for pelargonidin precursors c_m_ = 7.8 × 10^−9^, 7.8 × 10^−8^, 7.8 × 10^−7^). Indole was used at each concentration level in molar ratios of 1:1 and 5:1 compared to the pelargonidin glycoside. All combinations were carried out with three replicates (for one data point in treatment O2, 0.1 mg mL^−1^, ratio 1:5, 24 h, two replicates had to be removed). Due to a low solubility of indole in H_2_O, samples were vortexed and sonicated for a few seconds. In the case of 1.0 and 10.0 mg mL^−1^, aliquots were taken from the tubes and diluted with water (0.1% HCOOH [*v*/*v*]) to 0.1 mg mL^−1^ related to the pelargonidin glycoside at the following time points: 0.1, 1, 4, and 24 h. Directly afterwards, they were analysed by HPLC-PDA-HRESIMS.

For HPLC-PDA-HRESIMS, an Agilent Infinity 1260 HPLC equipped with a G1315D photodiode array detector was coupled to a Bruker Compact OTOF mass spectrometer (Bruker Daltonics, Bremen, Germany), controlled by the Bruker Compass control suite (version 1.9, Bruker Daltonics) together with Bruker OTOF Control version 4.0 (Bruker Daltonics). The samples were analysed in the positive ionisation mode in the mass range *m*/*z* 50 to 1300 using 30,000 m Δm^−1^ resolving power. The HPLC was equipped with a ZORBAX SB-C18 column (Agilent), 4.6 × 155 mm, particle diameter 3.5 µm. The flow was 0.5 mL min^−1^ with solvent A being H_2_O (0.1% HCOOH [*v*/*v*]) and solvent B MeOH (0.1% HCOOH [*v*/*v*]). Samples (4 µL) were injected during each run; 210, 254, 280, 350, 460, and 521 nm were used as detection wavelengths. The method used for pelargonidin 3-soph started with a focussing time of 1 min at 20% solvent B, followed by a gradient to 76% B within 14 min, and ended with a washing step (2 min gradient to 100% B, 4 min washing at 100% B, 2 min gradient back to starting conditions including 6 min equilibration time). The method used for orientalin consisted of a focussing time of 1 min at 5% solvent B, a gradient to 100% solvent B within 17 min, 3 min washing at 100% solvent B, 2 min gradient back to starting conditions and 6 min equilibration time. Data analysis and extraction of peak areas was carried out with Bruker compass data analysis 4.3.

## 4. Conclusions

Yellow *P. nudicaule* petals produce their pigments by a complex mechanism, combining the products of the flavonoid and tryptophan/indole biosynthetic pathways. The latter includes an indole-3-glycerol-phosphate lyase in the key step, which releases free indole and enables—with precise adjustment of reaction conditions—the final, unique biosynthetic step: the fusion of pelargonidin glycoside with indole. As the general pathway has recently been elaborated by retrobiosynthetic and precursor directed labelling studies [27,44], as well as biomimetic formation and synthetic studies [36,45], the present work identified the most important genes and their products involved in nudicaulin biosynthesis. Additionally, gene expression and metabolite abundance were reconstructed over five stages of petal development, during which the entire nudicaulin biosynthetic pathway takes place. DS 3 and 4 were identified as the most important stages, when gene expression of the flavonoid pathway and IGL is highest and the switch from precursor formation to nudicaulin production takes place. Crucial to this switch are an acidic vacuolar pH-value, a 7-*O*-glycosyl unit of pelargonidin in addition to the 3-*O*-glycosyl unit, and the reaching of a threshold concentration of both educts. The experimental verification of the recently proposed reaction mechanisms of the spontaneous fusion of indole and pelargonidin glycosides [27,45] including the structure confirmation of the intermediate detected in the current study would be interesting future targets. Prospective studies are not only motivated by the unprecedented cascade nature of the fusion reaction but also by the possibility of designing compounds with enhanced antiproliferative effects compared to some artificial nudicaulin derivatives [36].

The comparison of differently coloured *P. nudicaule* cultivars revealed that glycosylation of flavonoids at C7-OH is almost exclusively found in the nudicaulin producing yellow and orange cultivars, which are generally very similar in gene expression. However, three IGL candidates, putatively involved in indole production, show a significantly lower expression level in DS 5 in orange than in yellow flowers. This suggests that indole biosynthesis stops earlier in the orange cultivar, hence rendering indole the limiting factor in nudicaulin production. These IGL candidates are a good starting point for isolation and functional characterisation of the proteins responsible for the production of free indole in *P. nudicaule*. This knowledge could contribute to the identification of structure–function relationships of IGL proteins.

The chemical structure of the nudicaulins is very different from other yellow flower pigments [35], and, as hitherto known, their occurrence is restricted to the petals of only three species of Papaveraceae [31]. In *P. nudicaule*, nudicaulins occur in the apical petal areas, while yellow carotenoids and flavonols colour the pollen, stamens, and basal petal parts [28]. Hence, the flowers of this plant require three different biosynthetic pathways to produce yellow pigments. Among them, biosynthesis of nudicaulins appears to be most costly due to their nitrogen content. Thus, nudicaulins must have benefits for the plant that pay off the costs of their production. In general, flower colour is one important trait to attract pollinators, and is therefore crucial for reproductive success. Yellow flowers are, for example, preferred by some flies [19], which are predominant pollinators at high altitudes [67]. Additionally, compared to white, yellow petal colour results in elevated flower temperature, which helps pollinators to increase body temperature in cold regions and may enable better seed development by heated stigmata and ovaries [68]. As *P. nudicaule* was originally distributed in mountainous regions of Siberia and the steppes of Mongolia with a continental climate [32,33,34], temperature effects by yellow coloured petals could be an advantageous trait, both for pollination and in a physiological context. This is in accord with the prevailing occurrence of the yellow variety in nature. Moreover, flower colour is usually combined with olfactory stimuli which are especially important for long-distance signalling in habitats with dense vegetation and for naїve bees [69]. In *P. nudicaule*, colour and scent are biosynthetically linked because indole is an important precursor for nudicaulins and additionally released as scent component of yellow flowers [48]. Indole is not only emitted by *P. nudicaule* flowers, but is also present in the bouquet of many different plant families [70,71]. It is mainly attractive to hawkmoths [72,73] and flies [74], but also triggers a positive response in the oligolectic grey-backed mining bee *Andrena vaga* [75]. Furthermore, we have shown recently that honeybees are able to discriminate between artificial odour mixtures resembling the flower bouquets of *P. nudicaule* cultivars and differing in the presence or absence of indole [48]. Thus, the combination of colour and scent in yellow *P. nudicaule* flowers probably constitutes a unique characteristic recognised by pollinators.

It is also conceivable that nudicaulins or indole additionally play a role in herbivore defence, e.g., as feeding deterrents (probably such as the yellow pigments in *Hypericum calycinum* flowers, [1]) or signalling compounds (such as in maize [49] or rice [39]), respectively. Furthermore, functions in stress responses (e.g., UV light, cold) cannot be ruled out, although the presence of flavonols, which are known to have antioxidant and radical scavenging activity, might already have a protective effect. Ecological studies in the natural habitat of *P. nudicaule* including pollinator preferences, possible herbivores, or abiotic stresses would give decent insight into the benefits plants gain from the presence of nudicaulins in flowers.

Taken together, nudicaulins are fascinating natural products whose biosynthesis combines two pathways by the introduction of a TSA-homologous IGL enzyme providing free indole and spontaneous fusion of the educt molecules. Nudicaulins do not only testify to the stunning solution *P. nudicaule* has found to produce pigments and attract pollinators, but also are the basis to design new synthetic substances with interesting bioactive properties.

## Data Availability

For data availability, see Section 3.3.3 and Section 3.5.3.

## Supplementary Materials

The following are available online at https://www.mdpi.com/article/10.3390/ijms22084129/s1, Figure S1: structures of flavonol glycosides, present in *Papaver nudicaule* petals; Figure S2: structures of pelargonidin glycosides, present in *P. nudicaule* petals; Figure S3: structures of nudicaulins; Figure S4: diversification of tryptophan synthase alpha/indole-3-glycerol-phosphate lyase in *P. nudicaule* and other plant species; Figure S5: sequence alignment of TSA- and IGL-like proteins of *P. nudicaule* and other species; Figure S6: ^1^H NMR spectrum of pelargonidin 3-*O*-β-d-sophoroside; Figure S7: ^1^H-^1^H COSY spectrum of pelargonidin 3-*O*-β-d-sophoroside; Figure S8: HSQC spectrum of pelargonidin 3-*O*-β-d-sophoroside; Figure S9: HMBC spectrum of pelargonidin 3-*O*-β-d-sophoroside; Figure S10: HPLC-HR mass spectrum of pelargonidin 3-*O*-β-d-sophoroside; Figure S11: HPLC-HR mass spectrum of 7-deglucosylated intermediates I + II; Figure S12: HPLC-HR mass spectrum of 7-deglucosylated nudicaulins I + II; Figure S13: reaction of pelargonidin 3-*O*-β-d-sophoroside with indole. HPLC-MS base peak chromatogram and HPLC-PDA chromatogram; Figure S14: HPLC-HR mass spectrum of orientalin; Figure S15: HPLC-HR mass spectrum of intermediates I + II; Figure S16: HPLC-HR mass spectrum of nudicaulins I + II; Figure S17: cluster analysis of mRNA expression of DS 1–5 of yellow, orange, and white cultivars of *P. nudicaule*; Table S1: mRNA expression levels of nudicaulin/flavonoid biosynthetic pathway in DS 1–5 of white, yellow, and orange cultivars of *P. nudicaule*; Table S2: peak areas of metabolites involved in nudicaulin biosynthesis in DS 1–5 of yellow *P. nudicaule* flowers, identified by UPLC-HRMS/MS; Table S3: ^1^H NMR and ^13^C NMR spectroscopic data of pelargonidin 3-*O*-β-d-sophoroside; Table S4: final step of nudicaulin biosynthesis. HPLC-HRESIMS data of pelargonidin glycosides, intermediates, and nudicaulins; Table S5: peak areas of pigments in buds and flowers of four differently coloured cultivars of *P. nudicaule*, identified by UPLC-HRMS/MS; Table S6: definition of DSs of yellow *P. nudicaule* flowers. HPLC-PDA peak areas; Methods S1: 2D Differential gel electrophoresis
