# Supplementary material for: An Integrated—Omics/Chemistry Approach Unravels Enzymatic and Spontaneous Steps to Form Flavoalkaloidal Nudicaulin Pigments in Flowers of Papaver nudicaule L
Source: Int J Mol Sci. 2021 Apr 16;22(8):4129. doi: 10.3390/ijms22084129 (PMC8073789; doi:10.3390/ijms22084129)
Supplement: Supplementary file 1 [file ijms-22-04129-s001.zip › ijms-1155903-SI/210415_SI_Figures S1-S14_Tables S1-S5.pdf]

# An integrated –omics/chemistry approach unravels enzymatic and spontaneous steps to form flavoalkaloidal nudicaulin pigments in flowers of *Papaver nudicaule* L.

B. Dudek, A.-C. Warskulat, H. Vogel, N. Wielsch, R. C. Menezes, Y. Hupfer, C. Paetz, S. Gebauer-Jung, A. Svatoš, B. Schneider*

Max Planck Institute for Chemical Ecology, Jena, Germany

*For correspondence (email: schneider@ice.mpg.de)

## SUPPLEMENTARY MATERIALS

### FIGURES

page

**Figure S1.** Structures of flavonol glycosides, present in *Papaver nudicaule* petals. 2

**Figure S2.** Structures of pelargonidin glycosides, present in *P. nudicaule* petals. 3

**Figure S3.** Structures of nudicaulins. 4

**Figure S4**. Diversification of tryptophan synthase alpha/indole-3-glycerol-phosphate lyase in *P. nudicaule* and other plant species. 5

**Figure S5.** Sequence alignment of TSA- and IGL-like proteins of *P. nudicaule* and other species. 6

**Figure S6.** $^{1}$H NMR spectrum of pelargonidin 3-*O*-β-D-sophoroside. 7

**Figure S7.** $^{1}$H-$^{1}$H COSY spectrum of pelargonidin 3-*O*-β-D-sophoroside. 8

**Figure S8.** HSQC spectrum of pelargonidin 3-*O*-β-D-sophoroside. 9

**Figure S9.** HMBC spectrum of pelargonidin 3-*O*-β-D-sophoroside. 10

**Figure S10.** HPLC-HR mass spectrum of pelargonidin 3-*O*-β-D-sophoroside. 10

**Figure S11.** HPLC-HR mass spectrum of 7-deglucosylated intermediates I + II. 11

**Figure S12.** HPLC-HR mass spectrum of 7-deglucosylated nudicaulins I + II. 11

**Figure S13.** Reaction of pelargonidin 3-*O*-β-D-sophoroside with indole. HPLC-MS base peak chromatogram and HPLC-PDA chromatogram. 12

**Figure S14.** HPLC-HR mass spectrum of orientalin. 12

**Figure S15.** HPLC-HR mass spectrum of intermediates I + II. 13

**Figure S16.** HPLC-HR mass spectrum of nudicaulins I + II. 13

**Figure S17.** Cluster analysis of mRNA expression of DSs 1-5 of yellow, orange, and white cultivars of *P. nudicaule*. 14

### TABLES

**Table S1.** mRNA expression levels of nudicaulin/flavonoid biosynthetic pathway in DSs 1-5 of white, yellow, and orange cultivar of *P. nudicaule.* 15

**Table S2.** Peak areas of metabolites involved in nudicaulin biosynthesis in DSs 1-5 of yellow *P. nudicaule* flowers, identified by UPLC-HRMS/MS. 16

**Table S3.** $^{1}$H NMR and $^{13}$C NMR spectroscopic data of pelargonidin 3-*O*-β-D-sophoroside. 18

**Table S4.** Final step of nudicaulin biosynthesis. HPLC-HRESIMS data of pelargonidin glycosides, intermediates, and nudicaulins. 18

**Table S5.** Peak areas of pigments in buds and flowers of four differently coloured cultivars of *P. nudicaule*, identified by UPLC-HRMS/MS. 19

**METHODS**

**Methods S1.** 2D Differential gel electrophoresis 20

**Figure S1.** Structures of flavonol glycosides, present in *Papaver nudicaule* petals.

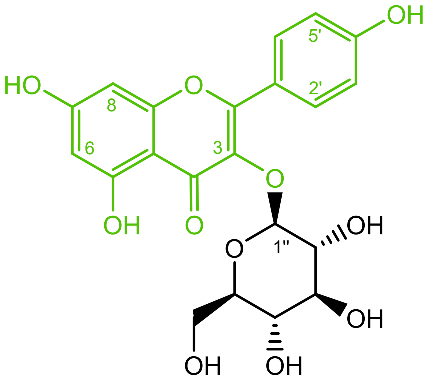

**Kaempferol 3-*O*-β-D-glucoside**
(kaempferol 3-glc)

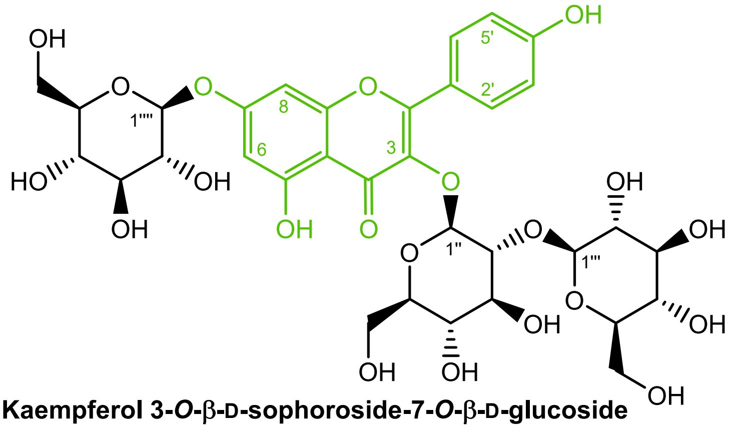

**Kaempferol 3-*O*-β-D-sophoroside-7-*O*-β-D-glucoside**
(kaempferol 3-soph-7-glc)

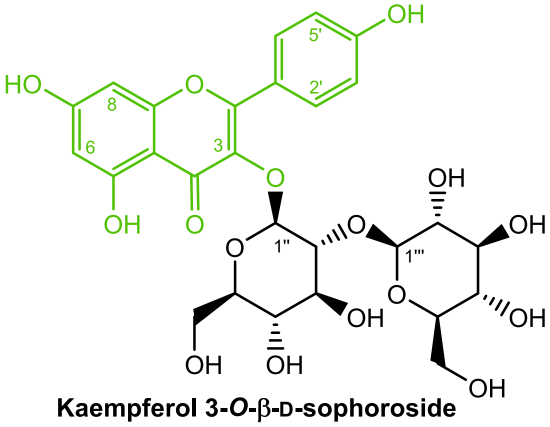

**Kaempferol 3-*O*-β-D-sophoroside**
(kaempferol 3-soph)

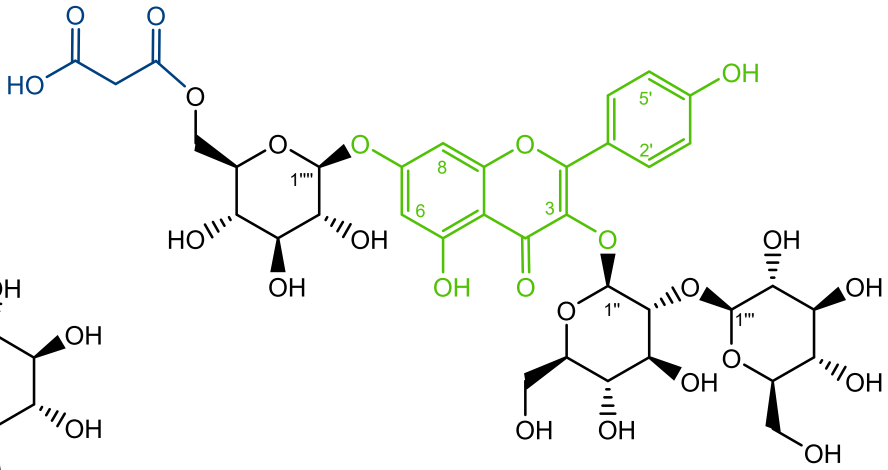

**Kaempferol 3-*O*-β-D-sophoroside-7-*O*-β-D-[(6-*O*-malonyl)glucoside]**
(kaempferol 3-soph-7-malglc)

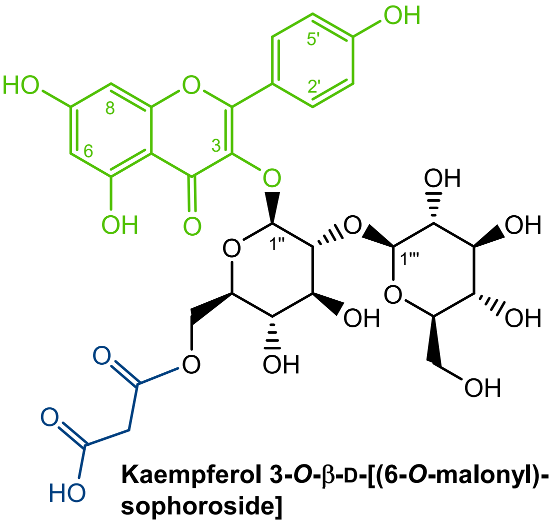

**Kaempferol 3-*O*-β-D-[(6-*O*-malonyl)-sophoroside]**
(kaempferol 3-malsoph)

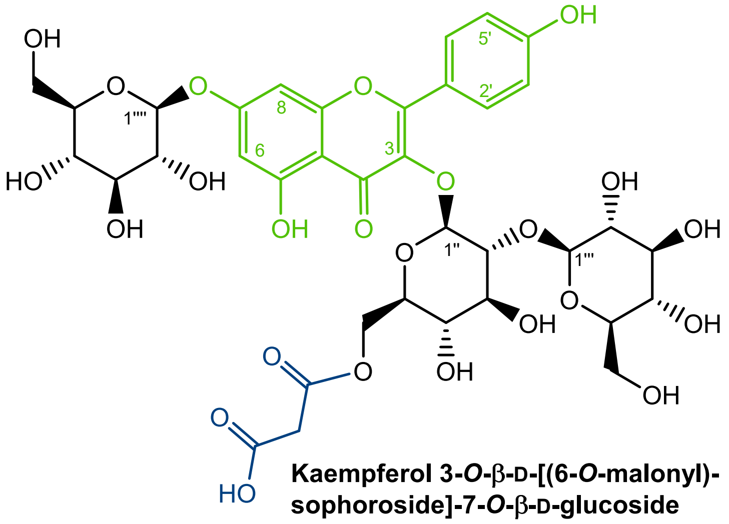

**Kaempferol 3-*O*-β-D-[(6-*O*-malonyl)-sophoroside]-7-*O*-β-D-glucoside**
(kaempferol 3-malsoph-7-glc)

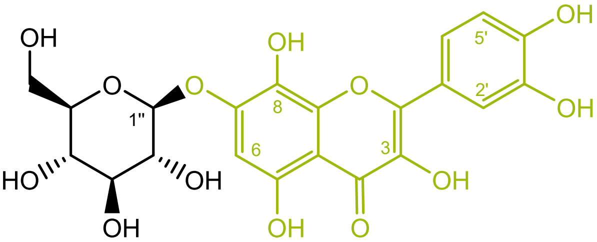

**Gossypetin 7-*O*-β-D-glucoside**
(gossypitrin)

**Figure S2.** Structures of pelargonidin glycosides, present in *P. nudicaule* petals. $X^{\ominus}$ represents counter ion of unspecified identity

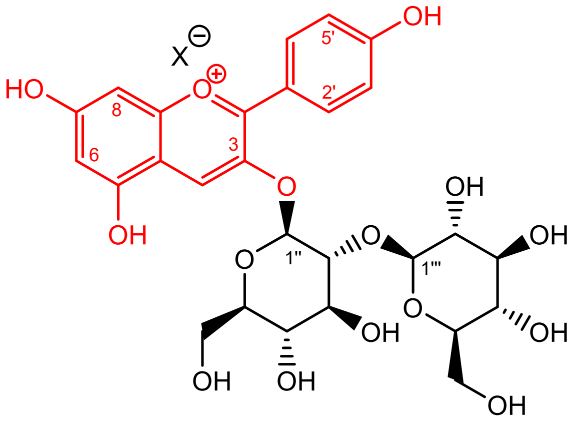

**Pelargonidin 3-*O*-β-D-sophoroside**
(pelargonidin 3-soph)

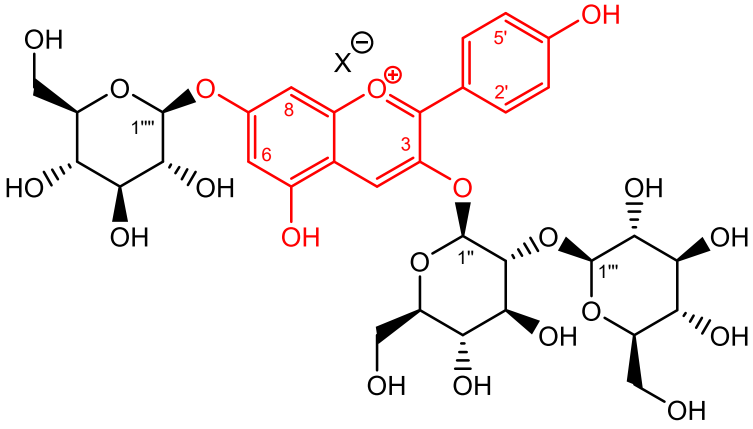

**Pelargonidin 3-*O*-β-D-sophoroside-7-*O*-β-D-glucoside**
(orientalin)

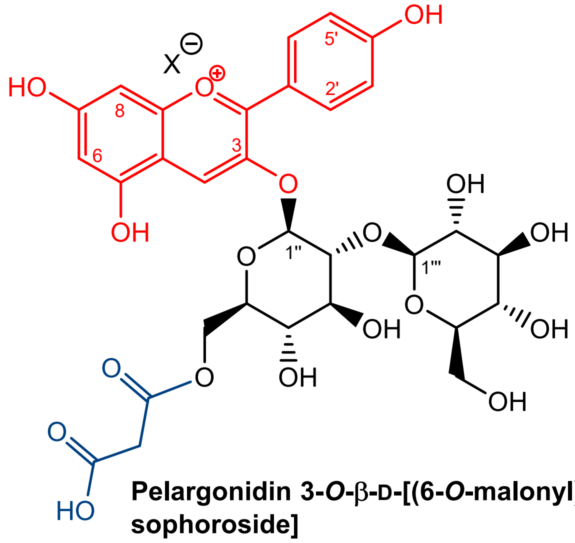

**Pelargonidin 3-*O*-β-D-[(6-*O*-malonyl)-sophoroside]**
(pelargonidin 3-malsoph)

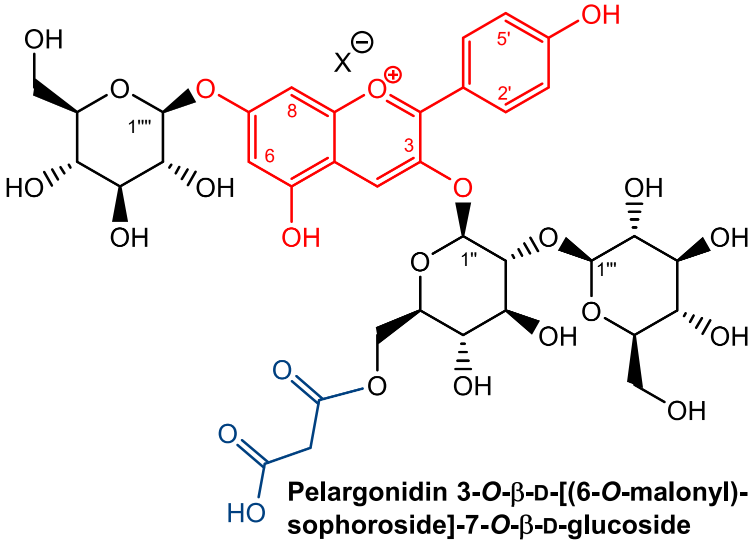

**Pelargonidin 3-*O*-β-D-[(6-*O*-malonyl)-sophoroside]-7-*O*-β-D-glucoside**
(pelargonidin 3-malsoph-7-glc)

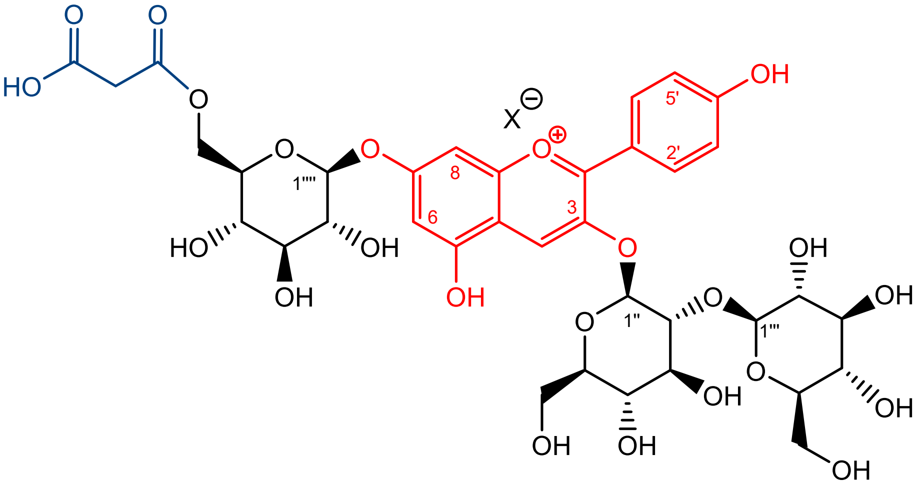

**Pelargonidin 3-*O*-β-D-sophoroside-7-*O*-β-D-[(6-*O*-malonyl)glucoside]**
(pelargonidin 3-soph-7-malglc)

**Figure S3.** Structures of nudicaulins (protonated forms). (a) Structures, present in *P. nudicaule* petals. The diastereomeric pairs nudicaulin III/IV, V/VI, VII/VIII have the same configuration as nudicaulins I/II. (b) DeGlc-nudicaulin I/II, produced synthetically from pelargonidin 3-*O*-β-D-sophoroside. $X^{\ominus}$ represents counter ion of unspecified identity.

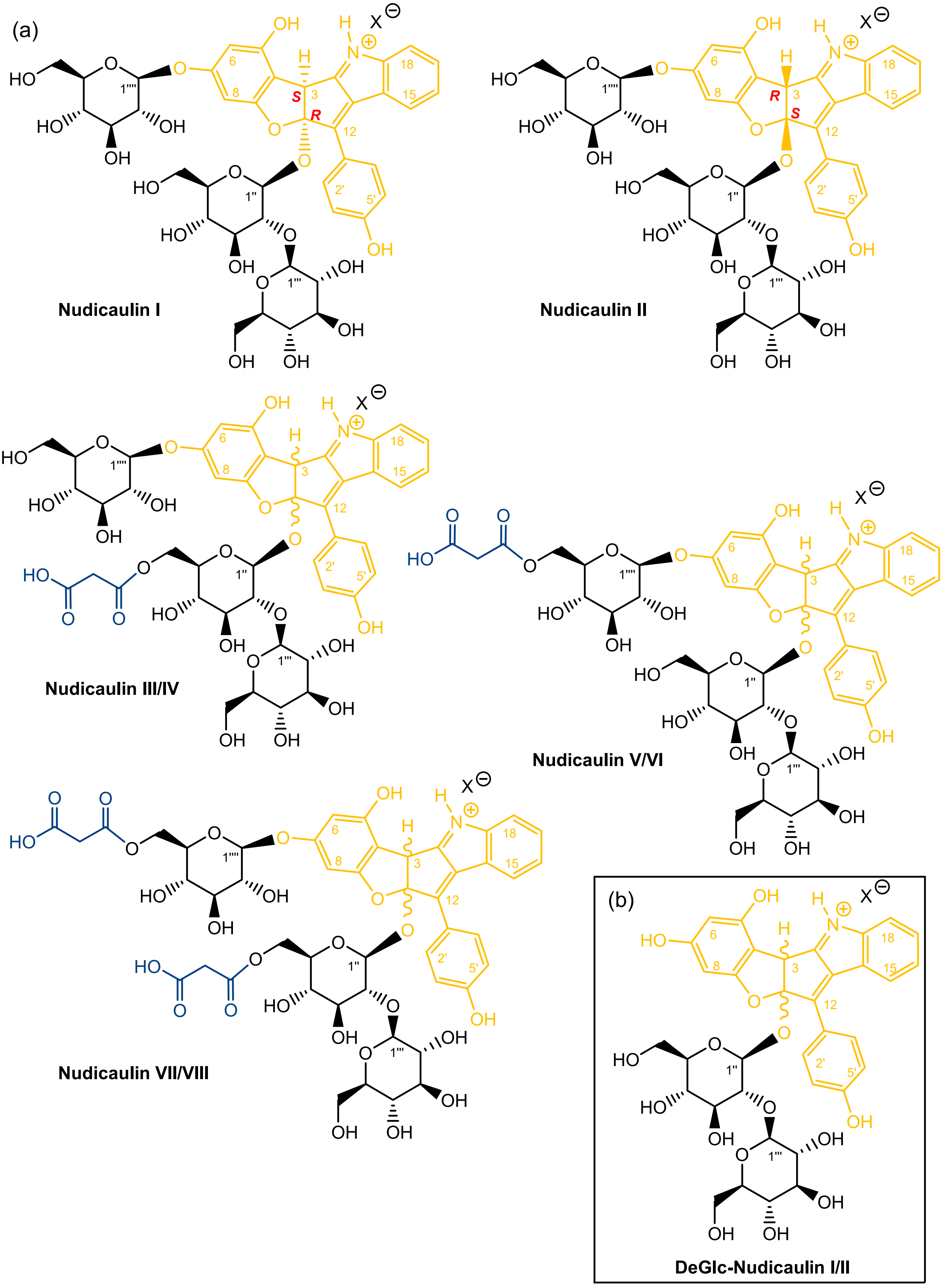

**Figure S4**. Diversification of tryptophan synthase alpha/indole-3-glycerol-phosphate lyase in *P. nudicaule* and other plant species. Maximum-likelihood inferred phylogeny of TSA and IGL-like predicted protein sequences identified in the *P. nudicaule* transcriptome and a subset of predicted plant TSA and IGL-like proteins. *P. nudicaule* sequences are highlighted with red letters. Likelihood-based support values are indicated on the corresponding nodes, and are shown for approximate likelihood-ratio test (SH-aLRT), Ultrafast bootstrap (1000 replicates) and the Approximate Bayes approach implemented in IQ-TREE version 1.6.12.

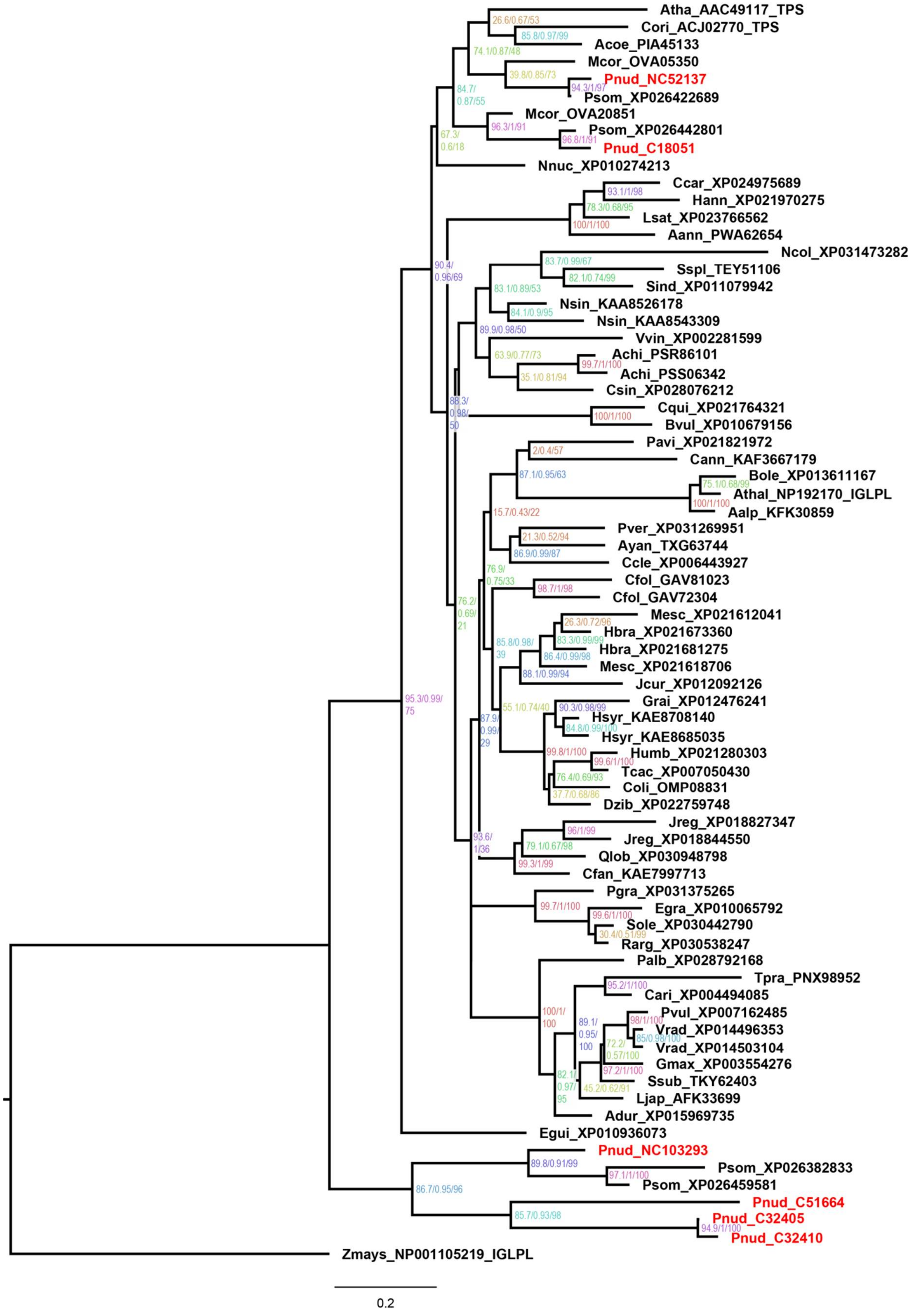

**Figure S5.** Sequence alignment of TSA- and IGL-like proteins of *P. nudicaule* and other species. The sequence of the *S. typhimurium* TSA is used as reference. Amino acids are coloured using the Clustal X colour scheme in Jalview. Species abbreviations are: Styp – *Salmonella typhimurium*, Zmays – *Zea mays*, Atha – *Arabidopsis thaliana*, Cori – *Consolida orientalis*, Psom – *Papaver somniferum*, Pnud – *Papaver nudicaule*, Bole – *Brassica oleracea* var. *oleracea*, Aann – *Artemisia annua*, Vvin – *Vitis vinifera*, Mesc – *Manihot esculenta*, Tcac – *Theobroma cacao*, Cfan – *Carpinus fangiana*, Gmax – *Glycine max*, Sole – *Syzygium oleosum*, Osati – *Oryza sativa*.

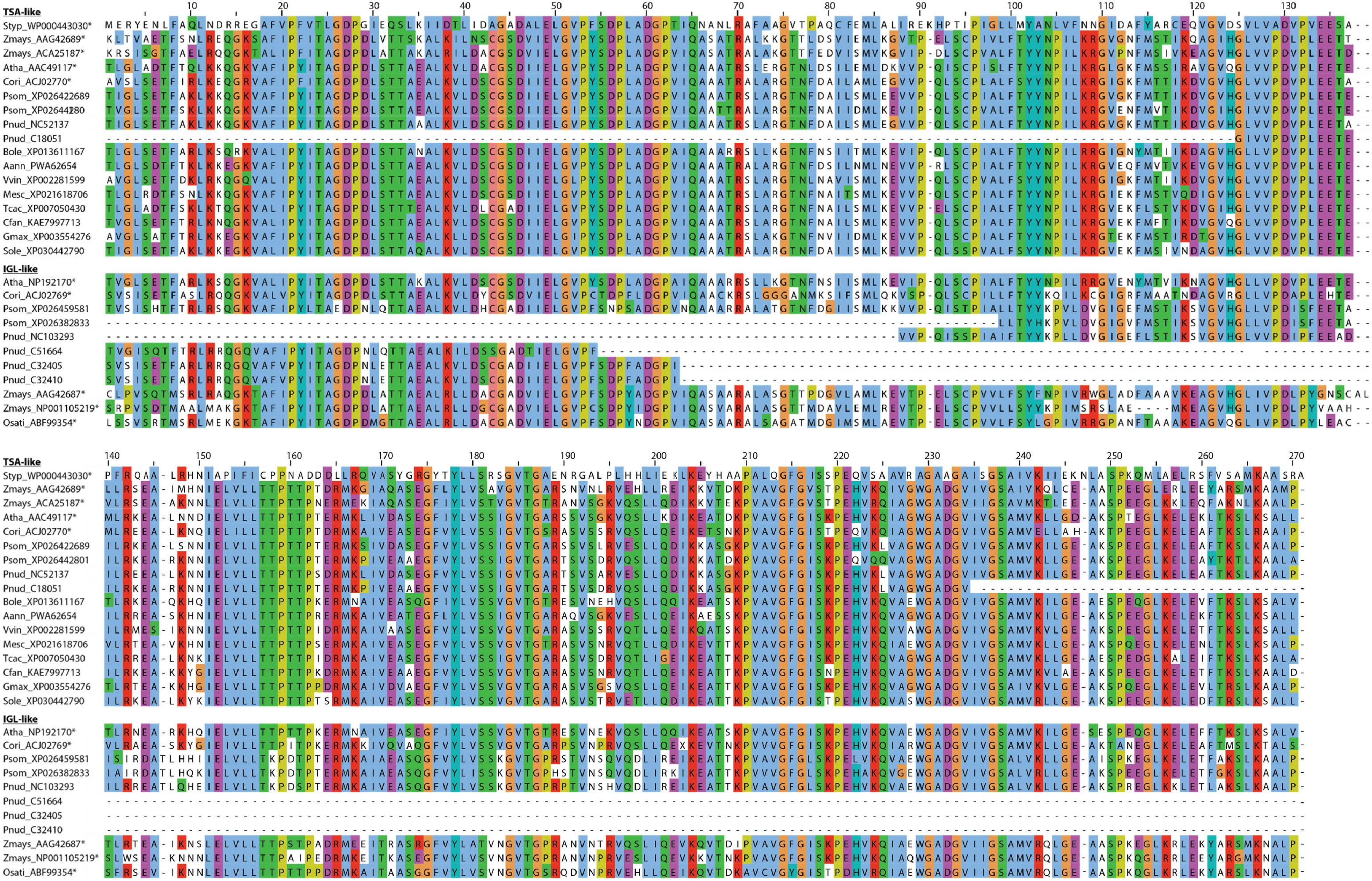

**Figure S6.** $^{1}$H NMR spectrum of pelargonidin 3-*O*-β-D-sophoroside (500 MHz, MeOH-$d_4$/ 2 % TFA-$d_1$).

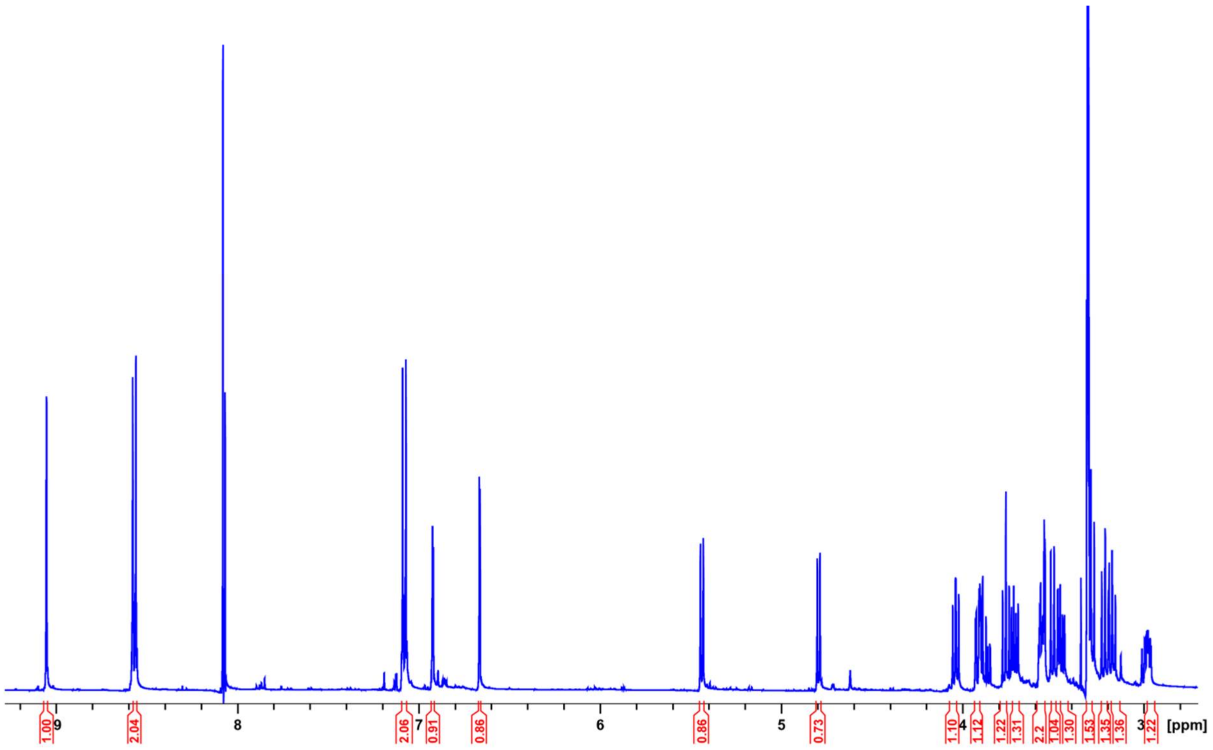

**Figure S7.** $^{1}H$-$^{1}H$ COSY spectrum of pelargonidin 3-*O*-β-D-sophoroside (500 MHz, MeOH-$d_4$/2 % TFA-$d_1$).

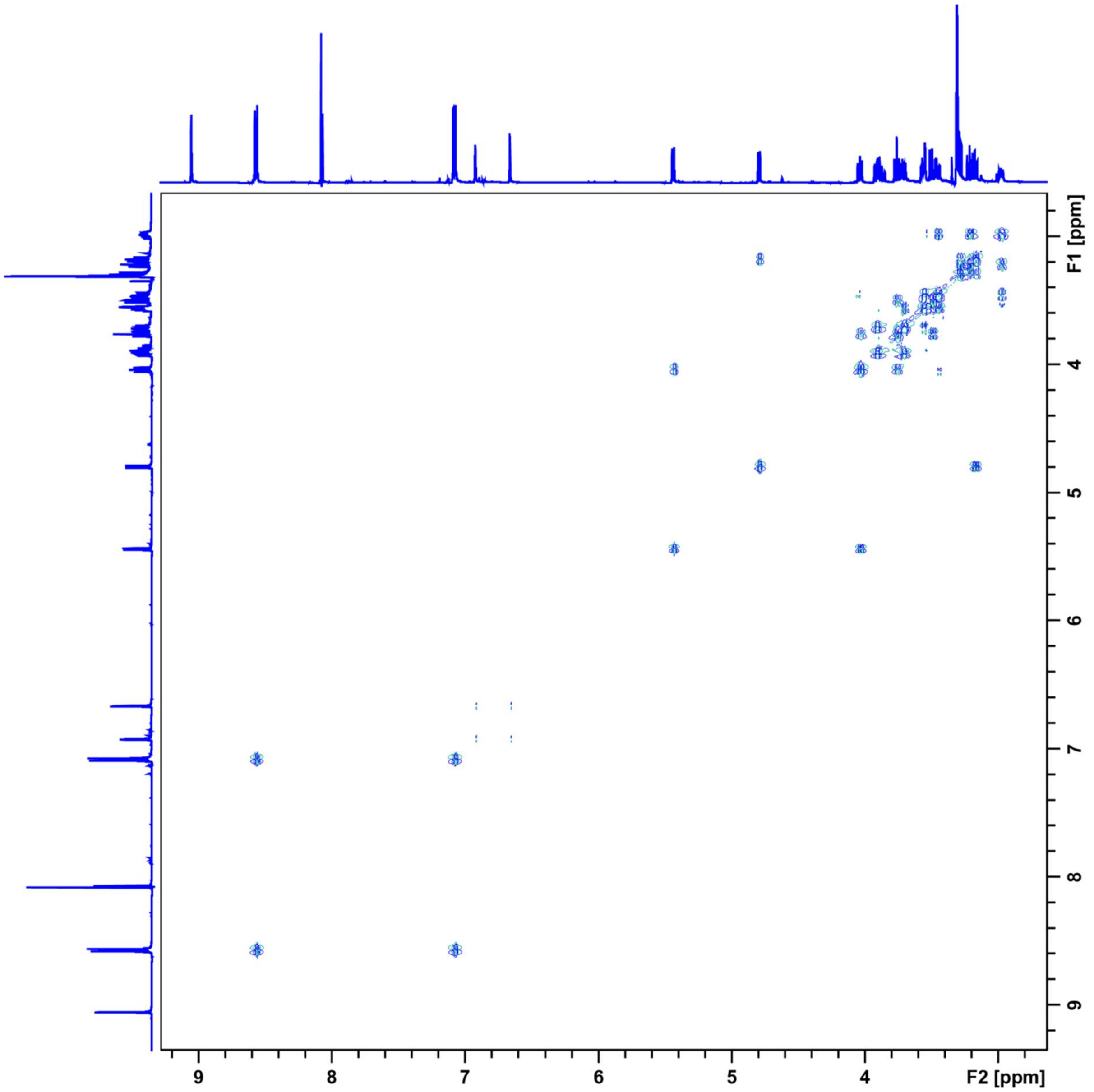

**Figure S8.** HSQC spectrum of pelargonidin 3-*O*-β-D-sophoroside (500 MHz, MeOH-$d_4$/ 2 % TFA-$d_1$).

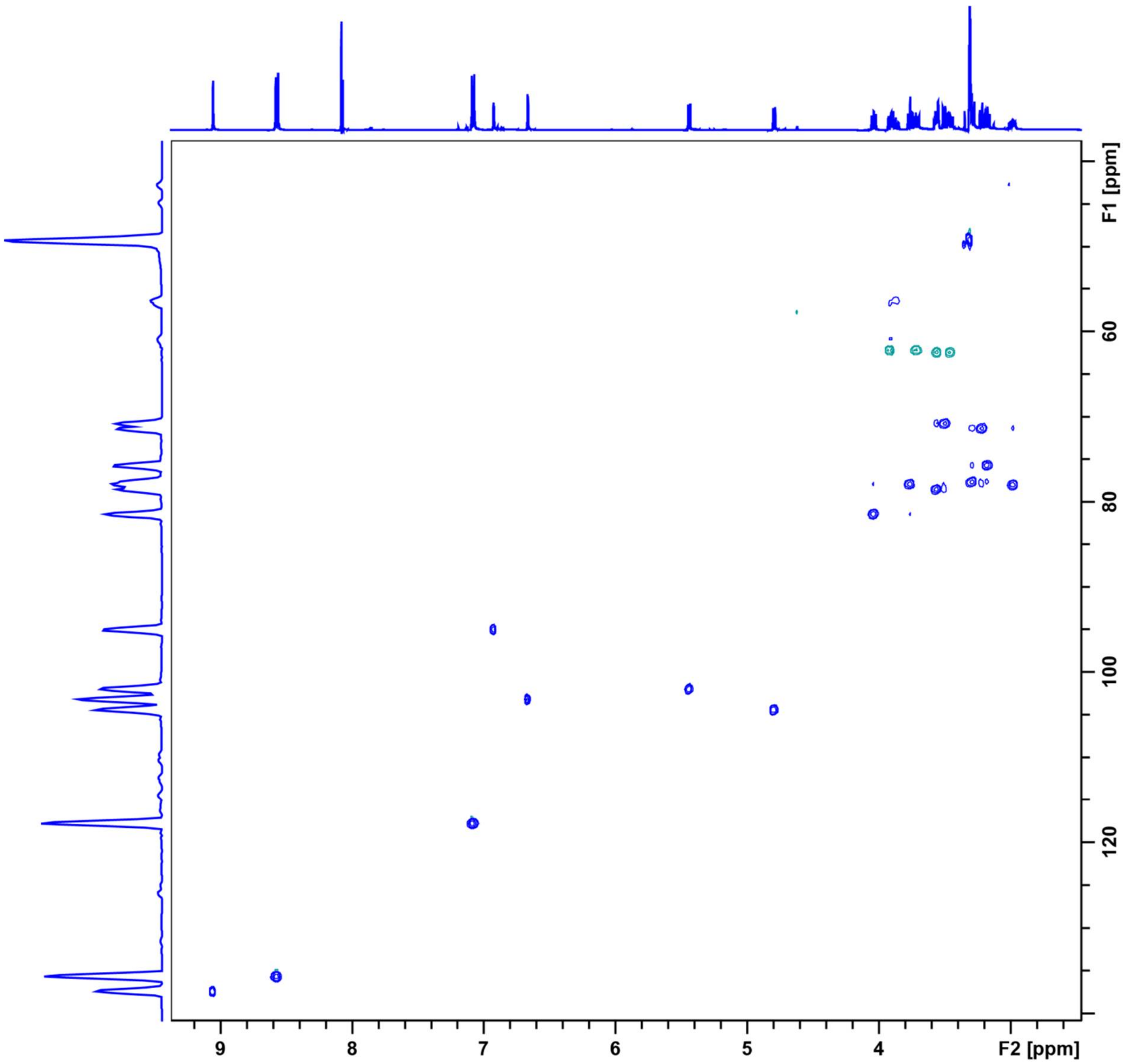

**Figure S9.** HMBC spectrum of pelargonidin 3-*O*-β-D-sophoroside (500 MHz, MeOH-$d_4$/ 2 % TFA-$d_1$).

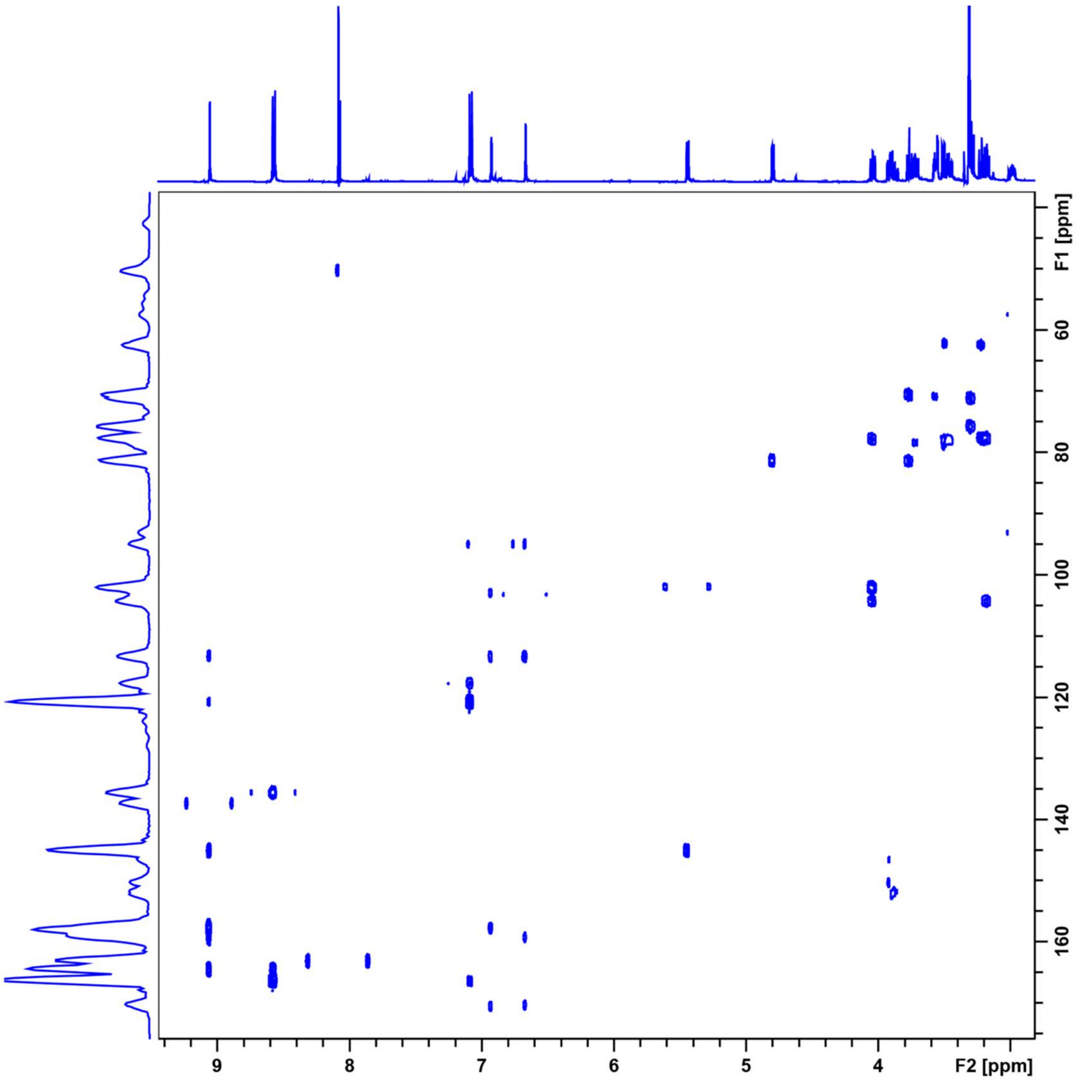


**Figure S10.** HPLC-HR mass spectrum of pelargonidin 3-*O*-β-D-sophoroside (positive ionisation mode).

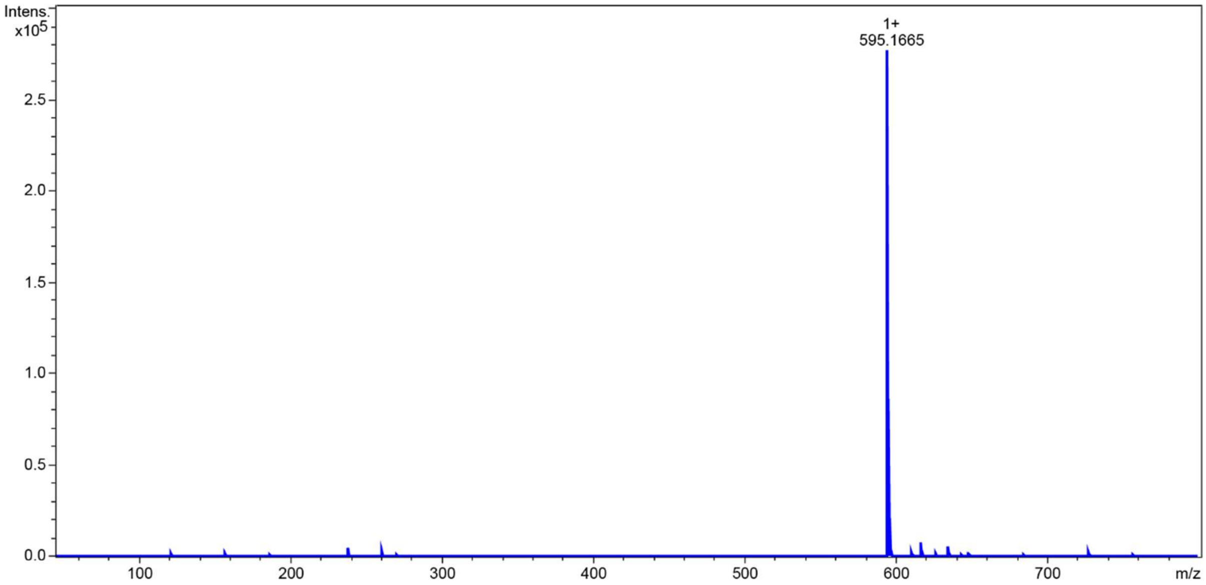

**Figure S11.** HPLC-HR mass spectrum of 7-deglucosylated intermediates **I** + **II**, derived from pelargonidin 3-*O*-β-D-sophoroside (positive ionisation mode).

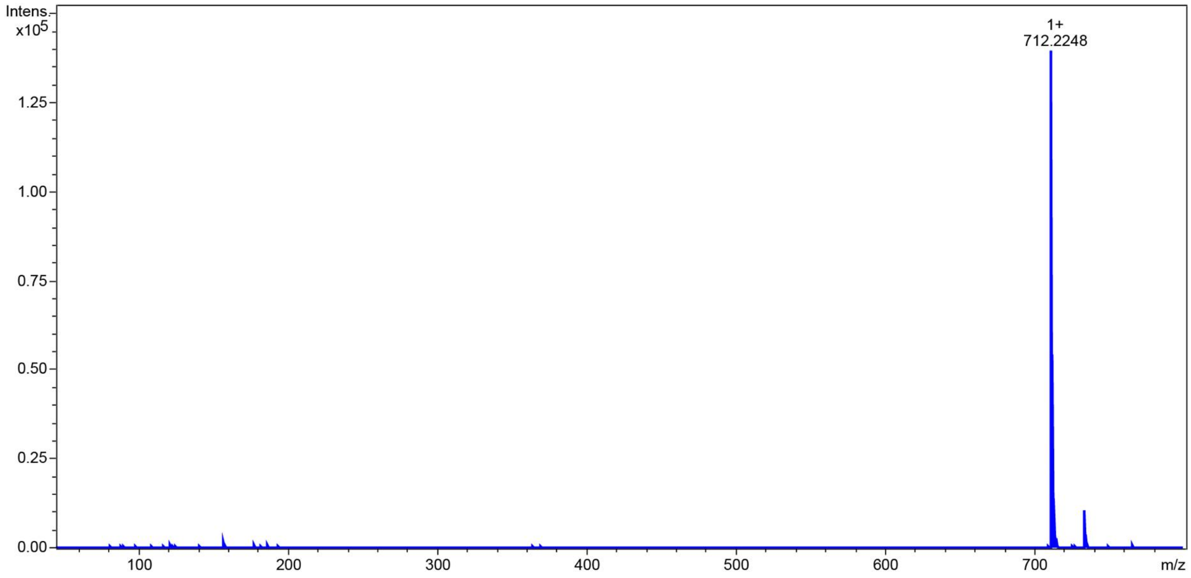


**Figure S12.** HPLC-HR mass spectrum of 7-deglucosylated nudicaulins **I** + **II**, derived from pelargonidin 3-*O*-β-D-sophoroside (positive ionisation mode).

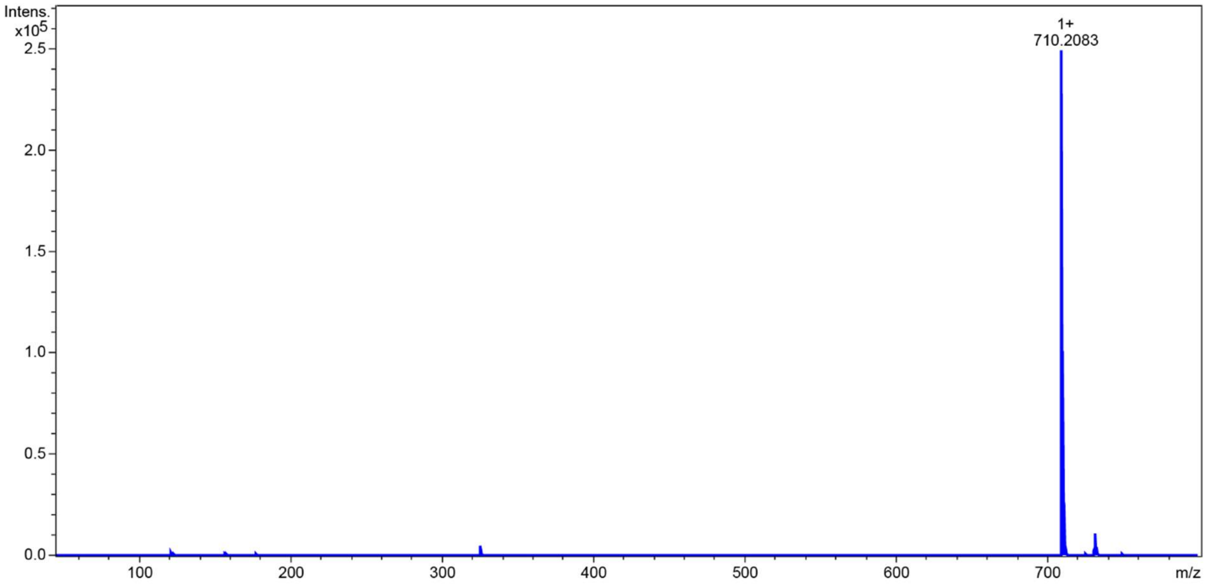

**Figure S13.** Reaction of pelargonidin 3-*O*-β-D-sophoroside with indole. HPLC-MS base peak chromatogram and HPLC-PDA chromatogram, 24 h after start of reaction ($c_P$ = 10 mg mL$^{-1}$, ratio 1:5, positive ionisation mode).

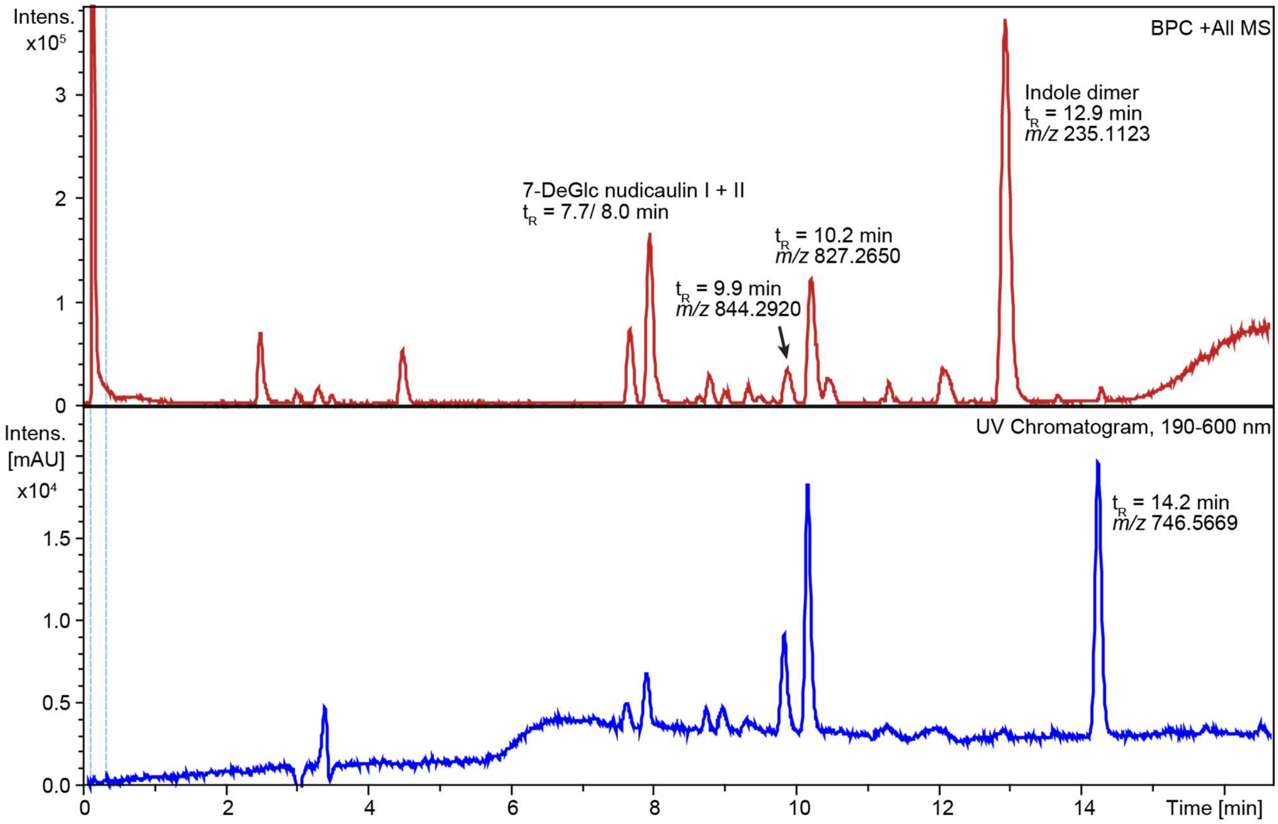


**Figure S14.** HPLC-HR mass spectrum of orientalin (positive ionisation mode).

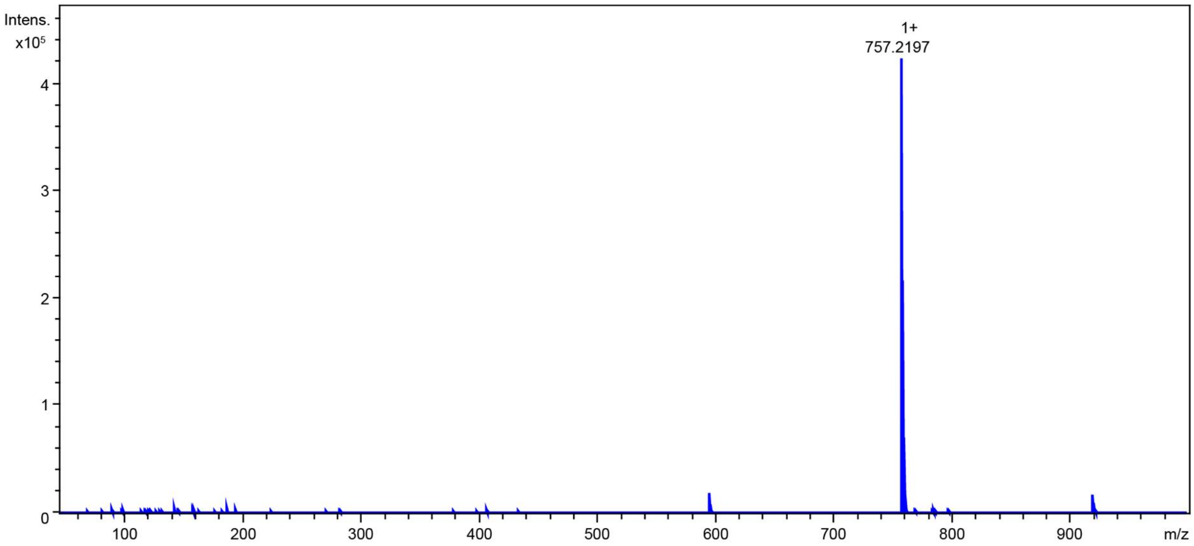

**Figure S15.** HPLC-HR mass spectrum of intermediates I + II, derived from orientalin (positive ionisation mode).

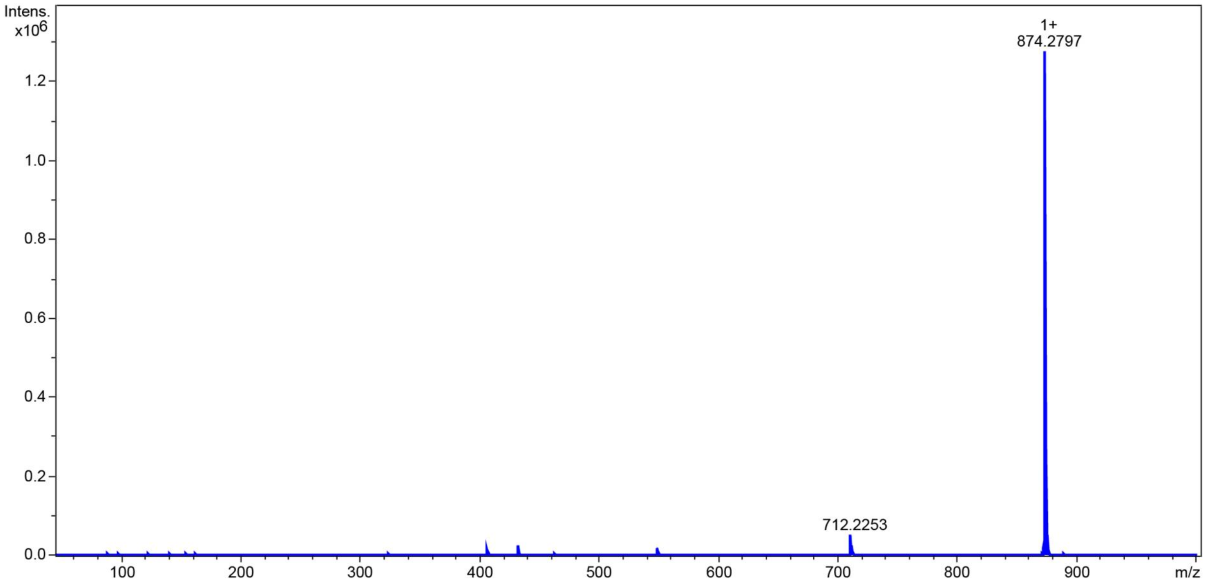


**Figure S16.** HPLC-HR mass spectrum of nudicaulins I + II, derived from orientalin (positive ionisation mode).

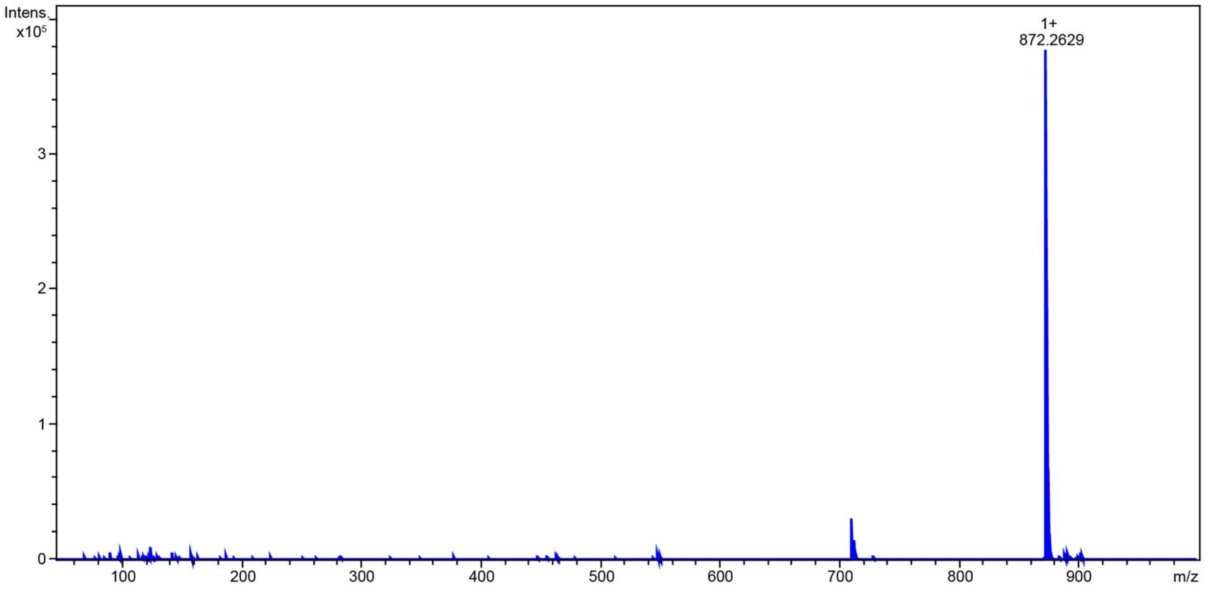

**Figure S17.** Cluster analysis of mRNA expression ($log_2$-transformed RPKM values, blue represents weakly-expressed genes, and red represents strongly-expressed genes) of developmental stages (DSs) 1-5 of yellow, orange, and white cultivars of *P. nudicaule.*

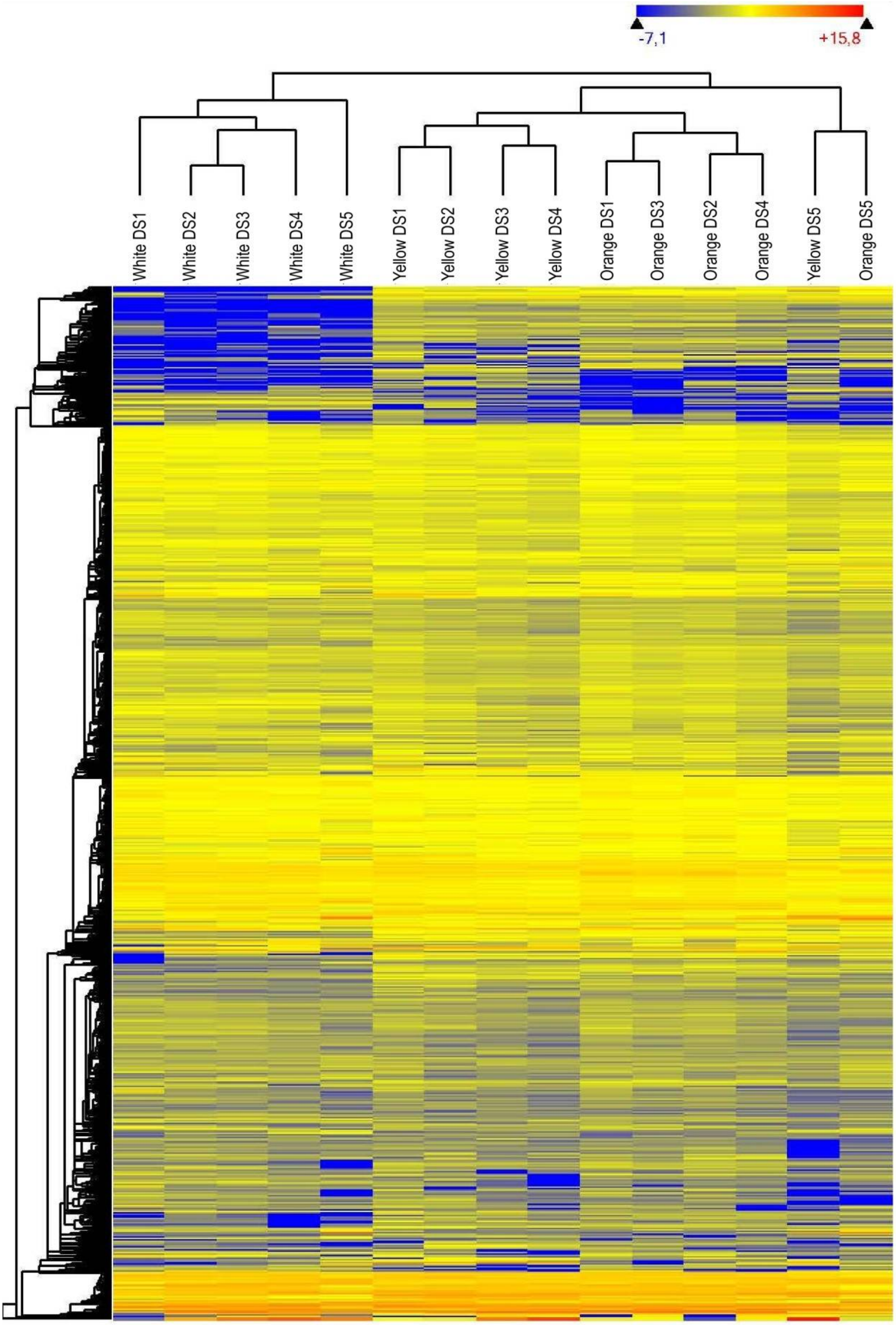

**Table S1.** mRNA expression levels ($log_2$-transformed RPKM values, blue represents weakly-expressed genes, and red represents strongly-expressed genes) of nudicaulin/ flavonoid biosynthetic pathway in developmental stages (DSs) 1-5 of white, yellow, and orange cultivar of *P. nudicaule.*

| Cultivar | White | | | | | Yellow | | | | | Orange | | | | |
|---|---|---|---|---|---|---|---|---|---|---|---|---|---|---|---|
| | DS1 | DS2 | DS3 | DS4 | DS5 | DS1 | DS2 | DS3 | DS4 | DS5 | DS1 | DS2 | DS3 | DS4 | DS5 |
| **Shikimate biosynthesis** | | | | | | | | | | | | | | | |
| **3-Deoxy-7-phosphoheptulonate synthase (EC 2.5.1.54)** | | | | | | | | | | | | | | | |
| C344 | 7.02 | 8.04 | 7.95 | 5.48 | 4.53 | 7.64 | 8.47 | 8.67 | 8.97 | 3.83 | 7.81 | 8.25 | 8.94 | 6.93 | 2.89 |
| C10293 | 4.75 | 5.88 | 5.23 | 4.31 | 1.90 | 4.71 | 4.27 | 5.35 | 5.35 | 2.82 | 5.27 | 5.36 | 4.95 | 4.03 | 2.94 |
| **3-Dehydroquinate synthase (EC 4.2.3.4)** | | | | | | | | | | | | | | | |
| **C65** | 5.33 | 6.73 | 6.70 | 6.69 | 5.82 | 6.31 | 6.83 | 7.09 | 7.24 | 6.61 | 6.85 | 6.98 | 7.14 | 6.92 | 5.70 |
| **Bifunctional 3-dehydroquinate dehydratase-shikimate dehydrogenase (EC 4.2.1.10, 1.1.1.25, 1.1.1.282)** | | | | | | | | | | | | | | | |
| **C612** | 3.94 | 5.06 | 4.58 | 3.98 | 2.67 | 4.59 | 4.74 | 4.48 | 4.10 | 3.63 | 5.14 | 5.26 | 5.10 | 4.18 | 3.63 |
| **Shikimate kinase (EC 2.7.1.71)** | | | | | | | | | | | | | | | |
| **C2405** | 4.43 | 4.22 | 4.12 | 4.49 | 3.93 | 5.42 | 6.27 | 7.02 | 7.23 | 4.55 | 4.21 | 4.30 | 5.13 | 4.48 | 3.43 |
| **5-Enolpyruvylshikimate-3-phosphate synthase (EC 2.5.1.19)** | | | | | | | | | | | | | | | |
| **C10589** | 6.37 | 7.99 | 7.68 | 5.93 | 1.32 | 6.57 | 7.08 | 7.66 | 7.87 | 4.44 | 7.62 | 7.61 | 7.97 | 6.46 | 2.63 |
| **Chorismate synthase (EC 4.2.3.5)** | | | | | | | | | | | | | | | |
| **C5454** | 0.38 | -1.71 | -2.97 | -0.99 | -0.01 | 6.20 | 2.11 | 1.72 | 5.66 | 5.59 | 6.60 | 6.88 | 6.31 | 6.03 | 5.10 |
| **C13704** | 6.25 | 7.15 | 6.85 | 6.41 | 5.18 | 6.17 | 6.70 | 6.89 | 6.55 | 5.86 | 6.96 | 7.09 | 7.05 | 6.56 | 4.98 |
| **Phenylalanine/Tyrosine biosynthesis** | | | | | | | | | | | | | | | |
| **Chorismate mutase (EC 5.4.99.5)** | | | | | | | | | | | | | | | |
| **C17568** | 5.02 | 4.97 | 4.44 | 3.73 | 3.30 | 7.08 | 7.27 | 6.61 | 5.82 | 6.13 | 5.55 | 5.58 | 4.93 | 4.71 | 4.14 |
| **Aminotransferase (EC 2.6.1.1, 2.6.1.5, 2.6.1.9, 2.6.1.57)** | | | | | | | | | | | | | | | |
| **C6952** | 5.75 | 6.04 | 5.94 | 5.50 | 5.02 | 5.26 | 5.74 | 5.56 | 5.82 | 4.96 | 5.86 | 6.02 | 5.71 | 5.78 | 4.65 |
| **C11591** | 6.58 | 5.86 | 5.62 | 5.14 | 2.78 | 5.18 | 5.30 | 5.21 | 4.85 | 2.96 | 5.67 | 5.34 | 5.31 | 4.65 | 2.54 |
| **C17184** | 5.24 | 6.22 | 6.53 | 7.24 | 6.44 | 5.74 | 5.94 | 5.95 | 6.33 | 7.25 | 6.13 | 6.03 | 6.46 | 6.73 | 7.05 |
| **Arogenate/prephenate dehydratase (EC 4.2.1.51, 4.2.1.91)** | | | | | | | | | | | | | | | |
| **C7313** | 2.28 | 4.44 | 4.45 | 3.75 | 2.46 | 4.93 | 5.25 | 5.41 | 5.37 | 2.83 | 4.75 | 4.49 | 5.80 | 3.67 | 3.26 |
| **C7314** | 4.21 | 5.27 | 5.58 | 5.19 | 4.00 | 6.96 | 6.53 | 6.17 | 6.48 | 3.76 | 4.16 | 3.34 | 6.25 | 5.32 | 4.83 |
| **C11712** | 3.28 | 3.94 | 3.70 | 3.41 | 2.86 | 4.36 | 4.62 | 3.81 | 3.74 | 3.42 | 3.82 | 3.70 | 3.88 | 3.61 | 2.73 |
| **Arogenate/prephenate dehydrogenase (EC 1.3.1.12, 1.3.1.13)** | | | | | | | | | | | | | | | |
| C5470 | 6.32 | 5.11 | 5.22 | 6.18 | 6.99 | 7.43 | 8.57 | 8.41 | 8.23 | 7.01 | 5.39 | 4.53 | 3.99 | 5.09 | 5.92 |
| C22441 | 3.46 | 3.76 | 3.14 | 2.58 | 0.11 | 3.62 | 3.84 | 2.52 | 2.69 | 2.09 | 3.54 | 3.09 | 2.97 | 2.18 | 0.92 |
| **Indole/Tryptophan biosynthesis** | | | | | | | | | | | | | | | |
| **Anthranilate synthase (EC 4.1.3.27)** | | | | | | | | | | | | | | | |
| C2332 | 4.08 | 5.14 | 4.97 | 4.58 | 3.38 | 4.79 | 5.08 | 5.53 | 5.07 | 3.68 | 5.30 | 4.86 | 5.65 | 5.18 | 3.47 |
| C17002 | -1.69 | -2.05 | -2.14 | 4.14 | 1.92 | 5.45 | 5.53 | 5.93 | 6.88 | 4.85 | 5.62 | 5.22 | 6.35 | 6.37 | 4.94 |
| C21664 | 2.90 | 4.25 | 4.76 | 4.17 | 1.52 | 2.91 | 2.69 | 4.85 | 5.61 | 5.58 | 3.26 | 4.22 | 5.89 | 5.65 | 6.87 |
| **Anthranilate phosphoribosyltransferase (EC 2.4.2.18)** | | | | | | | | | | | | | | | |
| C6276 | 5.55 | 4.90 | 4.68 | 5.18 | 3.29 | 4.05 | 4.46 | 4.36 | 3.60 | 3.22 | 4.89 | 4.80 | 4.13 | 3.91 | 2.90 |
| C17272 | 6.85 | 7.47 | 7.71 | 7.69 | 6.81 | 6.01 | 6.45 | 7.26 | 7.20 | 7.04 | 7.27 | 7.32 | 7.98 | 7.87 | 6.86 |
| **Phosphoribosylanthranilate isomerase (EC 5.3.1.24)** | | | | | | | | | | | | | | | |
| C19876 | 3.39 | 4.49 | 4.42 | 4.27 | 4.30 | 3.45 | 3.38 | 4.06 | 3.75 | 3.43 | 4.06 | 4.07 | 3.84 | 3.83 | 2.28 |
| **C32511** | 2.36 | 2.15 | 1.94 | 1.53 | 1.17 | 1.82 | 2.00 | -1.07 | -1.96 | -1.39 | 1.84 | 1.85 | 0.47 | 0.67 | 1.17 |
| **Indole-3-glycerol phosphate synthase (EC 4.1.1.48)** | | | | | | | | | | | | | | | |
| **C3186** | 5.93 | 6.50 | 6.89 | 6.95 | 5.62 | 5.92 | 6.17 | 6.79 | 6.69 | 6.77 | 6.26 | 6.60 | 6.87 | 7.36 | 6.63 |
| **Tryptophan synthase alpha & beta chain/indole-3-glycerol phosphate lyase (EC 4.2.1.20, 4.1.2.8)** | | | | | | | | | | | | | | | |
| **C485** | 6.03 | 6.93 | 6.92 | 6.51 | 4.86 | 5.69 | 5.99 | 6.31 | 6.29 | 5.61 | 6.11 | 6.50 | 6.24 | 6.15 | 4.51 |
| **NC52137** | 5.06 | 6.16 | 6.00 | 5.81 | 4.75 | 6.15 | 6.44 | 6.17 | 6.39 | 3.43 | 6.00 | 6.07 | 6.18 | 5.50 | 3.83 |
| **C18051** | 2.12 | 1.36 | 0.68 | 1.48 | -1.14 | 1.68 | 1.75 | 1.06 | 1.92 | 0.32 | 1.95 | 1.52 | 1.54 | 1.90 | -0.64 |
| NC103293 | -0.46 | 4.80 | 6.97 | 8.30 | -1.65 | 2.30 | 2.55 | 7.86 | 7.58 | 6.18 | 3.66 | 5.38 | 7.99 | 8.70 | 2.48 |
| **C32405** | -0.75 | 5.43 | 7.36 | 8.65 | -0.62 | 2.82 | 2.51 | 7.74 | 6.88 | 6.02 | 3.42 | 5.24 | 8.02 | 8.70 | 2.97 |
| **C32410** | -7.43 | -7.43 | -7.43 | -7.43 | -7.43 | -1.30 | 1.00 | 7.29 | 7.34 | 4.97 | 3.08 | 4.57 | 6.71 | 7.11 | 1.45 |
| **C51664** | -7.43 | -7.43 | -7.43 | -7.43 | -7.43 | -7.43 | -2.48 | 2.88 | -7.43 | -7.43 | -0.78 | -1.53 | 2.76 | 4.73 | 4.20 |
| **Phenylpropanoid biosynthesis** | | | | | | | | | | | | | | | |
| **Phenylalanine ammonia-lyase (EC 4.3.1.24, 4.3.1.25)** | | | | | | | | | | | | | | | |
| C1221 | 0.26 | 5.54 | 6.40 | 5.68 | 0.39 | 6.86 | 8.00 | 8.40 | 8.83 | 3.13 | 5.32 | 6.98 | 7.71 | 8.00 | 2.28 |
| C1222 | -0.15 | 6.42 | 7.21 | 5.83 | 1.78 | -1.18 | -2.77 | 7.90 | 7.51 | 4.20 | 5.65 | -1.82 | 7.92 | -1.78 | 3.58 |
| C2074 | 2.54 | 8.42 | 8.64 | 6.29 | 2.67 | 8.01 | 8.36 | 9.71 | 9.44 | 6.32 | 8.04 | 8.80 | 9.77 | 7.92 | 4.86 |
| **Cinnamate 4-hydroxylase (EC 1.14.14.91)** | | | | | | | | | | | | | | | |
| C16187 | 4.79 | 7.28 | 7.47 | 4.55 | -1.56 | 5.56 | 5.66 | 6.91 | 6.52 | 1.35 | 6.35 | 6.60 | 6.96 | 5.03 | 0.63 |
| **4-Coumarate-CoA ligase (EC 6.2.1.12)** | | | | | | | | | | | | | | | |
| C220 | 4.65 | 4.50 | 4.84 | 5.73 | 5.19 | 4.06 | 4.44 | 5.44 | 5.44 | 5.26 | 4.00 | 4.02 | 5.26 | 5.40 | 4.96 |
| C3630 | 4.81 | 5.93 | 6.37 | 8.16 | 8.43 | 5.94 | 5.92 | 7.04 | 7.89 | 9.51 | 6.06 | 5.86 | 7.15 | 8.55 | 9.19 |
| C5888 | 3.60 | 6.26 | 5.75 | 3.85 | 1.66 | 3.87 | 3.36 | 4.19 | 3.33 | 1.19 | 5.07 | 4.56 | 5.15 | 2.82 | 0.55 |
| C6994 | 1.86 | 7.21 | 7.15 | 5.19 | -1.76 | 6.22 | 6.90 | 8.85 | 8.39 | 4.34 | 7.09 | 7.93 | 8.28 | 7.80 | 3.04 |
| **Polyketide biosynthesis** | | | | | | | | | | | | | | | |
| **Acetyl-CoA carboxylase (EC 6.4.1.2)** | | | | | | | | | | | | | | | |
| **C1789** | 6.82 | 6.87 | 6.71 | 6.12 | 4.36 | 6.55 | 6.66 | 7.05 | 6.24 | 5.53 | 7.31 | 7.63 | 7.10 | 6.78 | 5.25 |
| **C7830** | 7.12 | 6.76 | 6.54 | 6.95 | 7.02 | 6.41 | 6.58 | 6.59 | 6.41 | 7.42 | 7.19 | 7.27 | 6.86 | 7.07 | 7.08 |
| **Flavonoid biosynthesis** | | | | | | | | | | | | | | | |
| **Chalcone synthase (EC 2.3.1.74)** | | | | | | | | | | | | | | | |
| **C2573** | 1.49 | 10.68 | 10.88 | 8.62 | 0.20 | 8.59 | 9.73 | 11.51 | 10.79 | 5.43 | 9.70 | 10.35 | 11.14 | 10.59 | 3.63 |
| **C9053** | -1.68 | 7.89 | 7.95 | 5.12 | -0.97 | 3.70 | 6.91 | 9.15 | 7.65 | -2.37 | 6.09 | 5.25 | 8.10 | 4.40 | -2.62 |
| **Chalcone-flavonone isomerase (EC 5.5.1.6)** | | | | | | | | | | | | | | | |
| **C336** | 1.51 | 9.26 | 8.45 | 5.60 | -0.72 | 8.87 | 8.68 | 8.86 | 8.45 | 0.86 | 9.50 | 9.66 | 8.97 | 8.04 | 0.54 |
| **C4358** | 6.07 | 8.40 | 7.84 | 8.20 | 8.87 | 7.43 | 7.28 | 7.38 | 7.34 | 8.87 | 7.99 | 7.75 | 7.62 | 8.53 | 8.68 |

| | | | | | | | | | | | | | | | |
|---|---|---|---|---|---|---|---|---|---|---|---|---|---|---|---|
| **Flavanone 3-hydroxylase (EC 1.14.11.9)** | | | | | | | | | | | | | | | |
| **C42** | 3.71 | 11.25 | 11.57 | 10.93 | 2.86 | 10.69 | 9.91 | 11.62 | 11.23 | 7.14 | 11.57 | 11.95 | 11.58 | 11.32 | 6.24 |
| **Flavonol biosynthesis** | | | | | | | | | | | | | | | |
| **Flavonol synthase (EC 1.14.20.6)** | | | | | | | | | | | | | | | |
| **C86** | 5.75 | 9.99 | 10.32 | 8.75 | 2.59 | 9.69 | 9.96 | 10.67 | 10.97 | 5.83 | 10.17 | 10.18 | 10.39 | 9.43 | 4.83 |
| **C8905** | 4.18 | 4.64 | 5.04 | 5.64 | 4.98 | 4.36 | 4.26 | 5.03 | 4.65 | 5.32 | 4.81 | 5.05 | 5.12 | 5.37 | 5.15 |
| **Anthocyanin biosynthesis** | | | | | | | | | | | | | | | |
| **Dihydroflavonol 4-reductase (EC 1.1.1.219)** | | | | | | | | | | | | | | | |
| **C138** | -0.19 | 7.92 | 6.75 | 0.72 | -2.65 | 9.06 | 8.66 | 9.79 | 10.03 | 8.06 | 10.11 | 11.17 | 10.61 | 10.92 | 1.72 |
| **C737** | 5.92 | 6.35 | 6.21 | 6.38 | 4.92 | 6.36 | 6.25 | 6.08 | 6.49 | 6.34 | 6.38 | 6.00 | 6.36 | 6.73 | 5.96 |
| **C5505** | 5.90 | 6.63 | 6.64 | 7.11 | 7.26 | 4.62 | 5.16 | 5.59 | 5.30 | 6.48 | 4.98 | 5.45 | 5.91 | 6.21 | 6.38 |
| **Anthocyanidin synthase (EC 1.14.20.4)** | | | | | | | | | | | | | | | |
| C73 | -1.22 | 4.30 | 5.04 | -0.35 | -4.42 | 9.72 | 9.61 | 10.95 | 10.26 | 5.15 | 9.60 | 10.72 | 10.16 | 10.33 | 2.71 |
| **C9486** | 2.31 | 2.47 | 2.67 | 3.59 | 4.55 | 2.79 | 2.39 | 2.52 | 2.54 | 3.16 | 2.71 | 2.51 | 2.76 | 2.45 | 3.72 |
| **C20733** | 2.40 | 8.13 | 10.06 | 11.33 | 9.79 | 5.21 | 5.34 | 4.32 | 5.31 | 4.89 | 5.22 | 7.12 | 6.91 | 8.41 | 8.94 |
| **C26642** | 4.34 | 4.83 | 3.53 | 5.14 | 0.88 | 2.76 | 2.79 | 5.53 | 3.66 | 2.60 | 3.55 | 3.16 | 4.09 | 4.55 | 4.89 |
| **Glucosyltransferases** | | | | | | | | | | | | | | | |
| **Glucosyltransferases (EC 2.4.1.115)** | | | | | | | | | | | | | | | |
| **C6023** | 0.99 | 8.27 | 9.38 | 3.60 | -0.15 | 7.99 | 8.51 | 11.12 | 10.78 | 8.07 | 9.78 | 10.41 | 11.25 | 11.30 | 1.71 |
| **C7130** | 0.30 | 8.23 | 9.25 | 2.70 | -3.04 | 8.16 | 8.33 | 11.06 | 10.56 | 7.57 | 10.08 | 10.70 | 11.19 | 11.07 | 2.39 |
| **C1482** | 1.09 | 6.29 | 6.49 | 4.64 | 0.86 | 8.05 | 8.51 | 9.78 | 9.57 | 4.70 | 8.94 | 9.89 | 9.87 | 8.59 | 2.30 |
| **C6854** | -4.84 | -7.06 | -3.28 | -7.06 | -1.54 | 5.85 | -2.55 | -1.05 | 5.02 | 3.51 | 4.06 | 4.90 | 5.07 | 4.88 | 1.22 |

**Table S2.** Peak areas of metabolites involved in nudicaulin biosynthesis in DS 1-5 of yellow *P. nudicaule* flowers, identified by UPLC-HRMS/MS (positive ionisation mode, three replicates each).

| Compound | | | Peak area | | | | | | | | | | | | | | |
|---|---|---|---|---|---|---|---|---|---|---|---|---|---|---|---|---|---|
| | *m/z* | RT (min) | DS 1-1 | DS 1-2 | DS 1-3 | DS 2-1 | DS 2-2 | DS 2-3 | DS 3-1 | DS 3-2 | DS 3-3 | DS 4-1 | DS 4-2 | DS 4-3 | DS 5-1 | DS 5-2 | DS 5-3 |
| 3-Dehydroshikimic acid | 173.0438 | 3.27 | 391,640 | 345,007 | 337,411 | 45,810 | 65,329 | 39,703 | 152,436 | 122,858 | 126,624 | 247,276 | 224,617 | 196,555 | 315,134 | 346,930 | 312,554 |
| Chorismic acid | 227.0587 | 7.42 | 176,735 | 323,057 | 270,473 | 54,464 | 84,960 | 81,314 | 67,463 | 51,860 | 66,781 | 55,272 | 47,826 | 63,945 | 44,423 | 221,923 | 28,225 |
| Anthranilic acid | 138.0543 | 3.18 | 1,791,831 | 3,569,049 | 2,353,822 | 1,296,084 | 3,609,816 | 3,110,189 | 5,162,113 | 2,608,573 | 9,328,778 | 4,940,938 | 2,006,567 | 5,135,679 | 5,034,915 | 3,299,194 | 3,762,159 |
| Tryptophan | 205.0962 | 11.99 | 779,398 | 884,793 | 658,161 | 174,588 | 480,534 | 643,751 | 556,637 | 452,911 | 785,732 | 636,397 | 645,484 | 831,039 | 1,222,537 | 883,944 | 1,337,667 |
| Phenylalanine | 166.0855 | 10.45 | 5,562,409 | 5,087,545 | 3,914,093 | 1,089,967 | 1,713,353 | 2,747,803 | 1,546,637 | 1,347,318 | 1,686,020 | 1,845,909 | 1,882,267 | 2,110,048 | 5,425,610 | 3,232,685 | 3,814,383 |
| Tyrosine | 182.0806 | 9.21 | 4,751,870 | 6,155,148 | 5,734,553 | 863,597 | 1,752,573 | 692,148 | 16,022 | 1,927,355 | 1,247,786 | 1,390,310 | 1,180,110 | 1,695,583 | 2,075,160 | 4,183,701 | 2,423,224 |
| trans-Cinnamic acid | 149.0588 | 12.18 | 31,710 | 69,418 | 26,358 | - | - | 11,022 | 84,536 | 137,979 | 411,403 | 1,768,175 | 1,516,937 | 1,358,298 | 1,825,971 | 2,058,006 | 2,063,740 |
| **Kaempferol** | | | | | | | | | | | | | | | | | |
| O-β-glucoside | 449.1069 | 16.48 | 260,014 | 430,521 | 525,636 | 117,010 | 146,304 | 192,052 | 150,769 | 124,575 | 311,480 | 516,429 | 661,290 | 591,586 | 379,320 | 278,891 | 469,241 |
| 3-O-β-sophoroside | 611.1602 | 14.87 | 758,254 | 1,223,604 | 1,540,719 | 195,082 | 503,411 | 391,212 | 448,134 | 381,936 | 467,595 | 599,741 | 682,286 | 574,662 | 596,750 | 540,068 | 627,869 |
| 3-O-β-[(6-malonyl)sophoroside] | 697.1611 | 15.38 | 5,248,732 | 7,535,149 | 7,138,802 | 2,451,233 | 3,411,965 | 2,439,900 | 3,315,176 | 1,791,980 | 4,352,152 | 4,114,307 | 4,135,245 | 5,005,685 | 2,610,634 | 3,815,575 | 1,777,400 |
| 3-O-β-[(6-malonyl)sophoroside]-7-O-β-glucoside | 859.2143 | 12.35 | 703,752 | 730,425 | 876,954 | 265,108 | 305,985 | 265,372 | 284,681 | 206,220 | 474,430 | 602,093 | 527,327 | 633,289 | 906,272 | 799,902 | 894,397 |
| 3-O-β-[(6-malonyl)sophoroside]-7-O-β-[(6-malonyl)glucoside] | 945.2158 | 13.34 | 1,540,654 | 1,067,504 | 1,962,405 | 856,688 | 1,085,924 | 504,864 | 2,397,693 | 1,683,507 | 3,256,369 | 3,933,885 | 3,780,129 | 3,914,310 | 3,897,078 | 4,621,971 | 3,661,131 |
| **Pelargonidin** | | | | | | | | | | | | | | | | | |
| 3-O-β-[(6-malonyl)sophoroside]-7-O-β-glucoside | 843.2192 | 11.34 | 1,860,423 | 2,260,108 | 2,306,738 | 1,939,303 | 1,560,617 | 1,879,816 | 3,671,679 | 2,460,882 | 4,416,090 | 2,667,458 | 2,442,148 | 3,103,189 | 376,245 | 969,278 | 243,537 |
| 3-O-β-[(6-malonyl)sophoroside]-7-O-β-[(6-malonyl)glucoside] | 929.2206 | 12.41 | 793,401 | 2,385,248 | 4,315,847 | 3,418,974 | 3,474,738 | 2,938,004 | 9,117,405 | 6,262,058 | 12,057,340 | 8,194,843 | 8,533,776 | 9,114,079 | 1,049,414 | 3,174,382 | 603,697 |
| Nudicaulin III & IV | 958.2624 | 15.50 | - | 8,533 | - | - | 7,258 | - | - | - | - | 4,741,889 | 2,998,778 | 3,990,282 | 7,591,219 | 7,255,899 | 8,829,110 |
| Nudicaulin VII & VIII | 1,044.2621 | 15.08 | 223,747 | 127,224 | 89,851 | 52,568 | 8,464 | - | 192,953 | 73,528 | 528,092 | 18,309,844 | 12,192,223 | 13,207,019 | 18,971,243 | 25,621,867 | 22,658,084 |
| Gossypetin 7-O-β-glucoside | 481.0968 | 15.11 | - | - | - | - | - | - | - | - | 522,140 | 2,158,475 | 1,411,097 | 2,806,360 | 3,030,353 | 2,665,820 | 2,658,546 |
| **Internal standards** | | | | | | | | | | | | | | | | | |
| [1-$^{13}$C]L-leucine | 133.1049 | 8.76 | 41,419,995 | 40,612,697 | 38,072,745 | 20,161,052 | 31,850,701 | 33,628,657 | 42,852,018 | 38,941,930 | 43,368,551 | 34,176,930 | 31,313,649 | 34,444,448 | 34,177,099 | 34,315,481 | 36,399,416 |
| Indole-3-propionic acid | 190.0854 | 19.00 | 13,472,980 | 15,114,417 | 14,513,631 | 3,758,319 | 5,685,060 | 7,324,928 | 7,684,970 | 7,060,437 | 8,299,127 | 9,239,596 | 9,311,290 | 9,869,602 | 9,870,491 | 10,526,754 | 10,939,743 |

**Table S3.** $^{1}H$ NMR and $^{13}C$ NMR spectroscopic data (500 MHz for $^{1}H$; 125 MHz for $^{13}C$) of pelargonidin 3-*O*-β-D-sophoroside in MeOH-$d_4$/TFA-$d_1$ 2 % (v/v).

| No. | $^{1}H$ δ, mult., *J* (Hz) | $^{13}C$ δ |
|---|---|---|
| **Aglycon** | | |
| **2** | | 164.4 |
| **3** | | 144.9 |
| **4** | 9.05, *s* | 137.4 |
| **5** | | 159.1 |
| **6** | 6.66, *d*, 2.0 | 103.1 |
| **7** | | 170.3 |
| **8** | 6.92, *d*, 2.0 | 95.0 |
| **9** | | 113.2 |
| **10** | | 158.0 |
| **1'** | | 120.7 |
| **2'/6'** | 8.57, *d*, 9.1 | 135.8 |
| **3'/5'** | 7.08, *d*, 9.1 | 117.7 |
| **4'** | | 166.3 |
| **Glucose A** | | |
| **1''** | 5.44, *d*, 7.6 | 101.0 |
| **2''** | 4.04, *dd*, 7.6, 8.8 | 81.3 |
| **3''** | 3.76, *dd*, 8.8, 8.9 | 77.8 |
| **4''** | 3.50, *dd*, 8.9, 8.9 | 70.8 |
| **5''** | 3.57, *m* | 78.5 |
| **6''A** | 3.72, *dd*, 12.2, 6.0 | 62.1 |
| **6''B** | 3.92, *dd*, 12.2, 2.2 | |
| **Glucose B** | | |
| **1'''** | 4.79, *d*, 7.7 | 104.4 |
| **2'''** | 3.18, *dd*, 7.7, 9.1 | 75.7 |
| **3'''** | 3.29, *dd*, 9.1, 9.1 | 77.6 |
| **4'''** | 3.22, *dd*, 9.1, 9.5 | 71.4 |
| **5'''** | 2.99, *ddd*, 9.5, 5.9, 2.5 | 78.0 |
| **6'''A** | 3.46, *dd*, 11.9, 5.9 | 62.5 |
| **6'''B** | 3.55, *dd*, 11.9, 2.5 | |

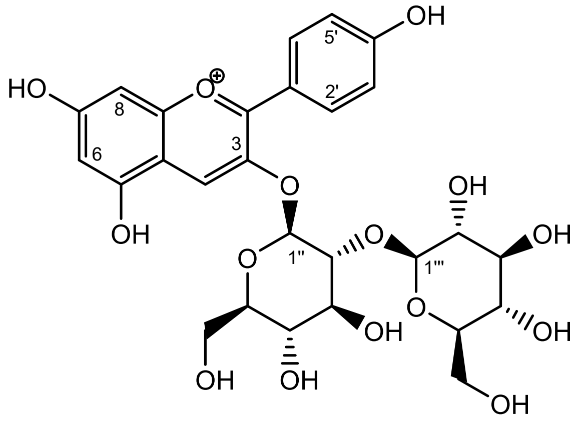


**Table S4.** Final step of nudicaulin biosynthesis. HPLC-HRESIMS data of pelargonidin glycosides, intermediates, and nudicaulins (positive ionisation mode).

| | ***m/z* [M-H]⁺** | |
|---|---|---|
| **compound** | **experimental** | **calculated (for)** |
| **Pelargonidin 3-*O*-β-D-sophoroside** | 595.1665 | 595.1657 ($C_{27}H_{31}O_{15}^{+}$ ) |
| **Intermediates a + b** | 712.2248 | 712.2236 ($C_{35}H_{38}NO_{15}^{+}$ ) |
| **7-Deglucosylated nudicaulins I + II** | 710.2083 | 710.2079 ($C_{35}H_{36}NO_{15}^{+}$ ) |
| **Orientalin** | 757.2197 | 757.2186 ($C_{33}H_{41}O_{20}^{+}$ ) |
| **Intermediates I + II** | 874.2797 | 874.2764 ($C_{41}H_{48}NO_{20}^{+}$) |
| **Nudicaulins I + II** | 872.2629 | 872.2608 ($C_{41}H_{46}NO_{20}^{+}$ ) |

**Table S5.** Peak areas of pigments in buds (Bu, developmental stage 3) and flowers (Fl) of four differently coloured cultivars of *P. nudicaule*, identified by UPLC-HRMS/MS (positive ionisation mode, three replicates each). Pigment aglycons are substituted with an *O*-β-D-glucosyl unit (glc), *O*-β-D-sophorosyl unit (soph), and/or –malonyl (mal) groups.

| Compound | Kaempferol- | | | | | | Pelargonidin- | | | | | Nudicaulin | | | Internal standard | |
|---|---|---|---|---|---|---|---|---|---|---|---|---|---|---|---|---|
| peak area | glc | 3-soph | 3-[(6-mal)-soph] | 3-soph-7-glc | 3-[(6-mall)-soph]-7-glc | 3-[(6-mal)-soph]-7-[(6-mal)glc] | 3-soph | 3-[(6-mal)-soph] | 3-soph-7-glc | 3-[(6-mal)-soph]-7-glc | 3-[(6-mal)-soph]-7-(6-mal)glc | Nudicaulin III & IV | Nudicaulin V & VI | Nudicaulin VII & VIII | [1-$^{13}$C]L-leucine | Indole-3-propionic acid |
| *m/z* | 449.1069 | 611.1595 | 697.1598 | 773.2123 | 859.2125 | 945.2128 | 595.1647 | 681.1647 | 757.2172 | 843.2175 | 929.2179 | 958.2595 | 958.2593 | 1044.2599 | 133.1051 | 190.0858 |
| RT (min) | 15.36 | 13.83 | 14.47 | 11.05 | 11.64 | 12.77 | 10.72 | 12.12 | 8.92 | 10.23 | 11.46 | 13.30 | 13.45 | 14.56 | 3.44 | 17.71 |
| White_Bu-1 | 80,725,573 | 266,494,358 | 178,239,030 | 7,850,008 | 1,364,516 | - | 5,879,006 | 6,785,439 | 82,791 | 86,682 | - | - | 50,500 | - | 2,497,162,160 | 814,853,958 |
| White_Bu-2 | 322,547,599 | 1,064,790,971 | 933,382,931 | 12,730,807 | 3,470,784 | - | 31,893,029 | 34,999,603 | 948,150 | 933,155 | - | - | - | - | 2,525,335,283 | 804,010,794 |
| White_Bu-3 | 182,702,479 | 713,172,488 | 593,755,384 | 14,240,562 | 2,524,286 | - | 27,229,298 | 29,875,916 | 514,452 | 394,269 | - | - | - | - | 2,407,980,128 | 707,012,144 |
| White_Fl-1 | 836,661,101 | 2,185,211,912 | 1,983,959,282 | 51,048,571 | 26,470,505 | 50,096 | 37,330,510 | 35,789,618 | 2,095,655 | 1,749,915 | 102,047 | 217,053 | 576,456 | 91,643 | 1,561,861,732 | 809,717,851 |
| White_Fl-2 | 579,078,408 | 1,697,799,198 | 1,506,017,309 | 44,196,510 | 22,239,341 | - | 45,009,406 | 49,612,994 | 3,299,593 | 2,469,311 | 122,859 | 71,279 | 194,115 | - | 1,121,059,965 | 761,293,457 |
| White_Fl-3 | 1,203,079,226 | 636,461,257 | 270,561,877 | 43,083,004 | 4,720,542 | - | 269,947 | 387,748 | 96,162 | - | - | - | - | - | 1,730,770,196 | 704,777,614 |
| Red_Bu-1 | 140,128,189 | 1,029,742,988 | 818,687,376 | 6,686,279 | 4,109,937 | 522,750 | 2,383,558,100 | 2,549,627,829 | 6,831,429 | 6,282,032 | 1,426,817 | 652,577 | 892,461 | 1,461,197 | 1,448,181,612 | 708,581,332 |
| Red_Bu-2 | 161,240,095 | 580,075,001 | 440,923,270 | 7,190,720 | 3,507,348 | 521,726 | 430,714,978 | 408,919,417 | 11,013,303 | 10,358,776 | 1,670,420 | 939,636 | 1,642,181 | 2,110,686 | 1,928,580,796 | 653,074,839 |
| Red_Bu-3 | 102,573,102 | 447,505,902 | 431,362,607 | 8,987,370 | 5,569,498 | 1,425,565 | 206,657,992 | 220,302,620 | 16,534,170 | 16,859,755 | 3,012,223 | 1,644,702 | 1,607,569 | 2,819,646 | 888,925,952 | 657,219,811 |
| Red_Fl-1 | 187,603,525 | 1,144,641,544 | 852,000,205 | 28,659,317 | 17,565,267 | 127,138 | 7,907,642,232 | 7,792,370,247 | 42,470,579 | 24,490,713 | 2,954,652 | 665,193 | 913,498 | 1,768,653 | 2,178,333,442 | 782,927,014 |
| Red_Fl-2 | 92,777,974 | 778,203,156 | 722,537,646 | 22,045,214 | 2,586,580 | 694,841 | 7,543,554,868 | 7,455,604,774 | 17,805,195 | 32,791,478 | 473,399 | 1,074,981 | 1,313,078 | 2,003,579 | 1,890,712,933 | 683,040,467 |
| Red_Fl-3 | 126,214,516 | 755,724,303 | 630,687,846 | 23,846,482 | 17,879,357 | 453,939 | 5,859,444,786 | 5,836,112,968 | 30,057,777 | 29,845,238 | 2,031,748 | 702,270 | 1,209,572 | 1,295,326 | 1,299,271,302 | 741,539,161 |
| Orange_Bu-1 | 310,108,101 | 458,926,099 | 271,431,145 | 53,018,748 | 32,616,957 | 6,036,125 | 37,261,555 | 14,910,612 | 188,717,047 | 158,341,616 | 14,464,027 | 159,051 | 241,047 | 256,502 | 1,639,805,195 | 850,226,868 |
| Orange_Bu-2 | 365,433,769 | 507,507,980 | 283,119,378 | 60,601,305 | 40,993,268 | 9,753,994 | 10,114,826 | 6,397,913 | 221,768,297 | 217,532,701 | 43,487,277 | - | 417,505 | 85,778 | 1,706,594,628 | 638,484,140 |
| Orange_Bu-3 | 203,220,428 | 678,453,913 | 398,450,437 | 148,122,141 | 107,000,366 | 22,868,768 | 16,395,323 | 5,206,391 | 302,604,693 | 366,128,462 | 63,456,980 | 256,684 | 364,442 | 321,146 | 1,901,616,162 | 594,880,731 |
| Orange_Fl-1 | 129,599,305 | 231,138,119 | 127,525,317 | 248,466,003 | 226,952,307 | 77,593,441 | 8,947,294 | 3,793,606 | 702,459,051 | 916,766,803 | 363,251,293 | 144,242,810 | 208,793,258 | 324,647,303 | 1,778,450,189 | 740,284,163 |
| Orange_Fl-2 | 26,220,311 | 145,105,884 | 83,566,158 | 209,111,097 | 141,807,961 | 9,681,716 | 2,731,284 | 2,158,271 | 884,462,977 | 742,885,155 | 902,063 | 51,460,279 | 71,606,079 | 1,439,261 | 1,520,432,867 | 640,147,429 |
| Orange_Fl-3 | 98,102,670 | 219,693,432 | 110,712,000 | 311,271,458 | 257,141,143 | 110,927,561 | 6,198,734 | 2,860,820 | 804,268,989 | 1,013,010,792 | 388,822,734 | 172,371,228 | 227,722,875 | 303,206,944 | 1,609,371,639 | 602,825,088 |
| Yellow_Bu-1 | 22,964,944 | 211,647,457 | 143,547,860 | 72,767,151 | 64,721,296 | 23,925,921 | 4,860,679 | 2,426,714 | 274,191,574 | 377,234,428 | 130,074,350 | 356,845 | 801,382 | 785,506 | 1,900,495,924 | 753,359,610 |
| Yellow_Bu-2 | 207,995,520 | 592,842,334 | 485,908,101 | 188,255,729 | 164,659,941 | 92,391,742 | 6,420,749 | 4,297,529 | 251,260,536 | 494,874,359 | 184,859,338 | 2,814,996 | 4,163,960 | 2,343,447 | 2,345,703,335 | 847,634,411 |
| Yellow_Bu-3 | 81,118,866 | 442,609,179 | 338,101,324 | 149,840,810 | 124,824,901 | 57,278,508 | 1,716,409 | 888,881 | 182,679,057 | 251,210,692 | 82,175,883 | 1,361,677 | 2,605,457 | 1,137,148 | 2,044,482,443 | 740,607,409 |
| Yellow_Fl-1 | 40,720,212 | 113,430,260 | 97,896,244 | 304,583,764 | 252,422,389 | 102,595,610 | 5,228,707 | 931,278 | 22,720,117 | 32,510,961 | 12,374,853 | 447,036,295 | 816,771,127 | 1,261,747,715 | 1,409,634,658 | 553,283,587 |
| Yellow_Fl-2 | 29,760,696 | 72,257,310 | 50,792,466 | 209,894,042 | 176,562,413 | 80,688,353 | 2,828,954 | 591,645 | 19,495,329 | 30,390,950 | 12,325,922 | 525,551,126 | 758,281,565 | 1,394,702,268 | 1,479,343,051 | 653,273,179 |
| Yellow_Fl-3 | 61,247,012 | 254,858,788 | 208,039,571 | 416,752,359 | 358,829,789 | 156,947,130 | 7,969,374 | 849,387 | 17,786,521 | 22,558,666 | 9,725,687 | 498,317,966 | 789,987,776 | 1,263,594,151 | 1,774,896,191 | 561,820,824 |

**Methods S1. 2D Differential gel electrophoresis (2D-DIGE)**

To enable a direct comparison between the different DSs, extracted protein samples were subjected to a 2D-differential gel electrophoresis (2D-DIGE), following the protocol of Wartenberg et al. (Wartenberg *et al.*, 2012). The Refraction-2D$^{TM}$ Labelling Kit (NH DyeAGNOSTICS GmbH, Halle, Germany) was used. Samples (50 µg) from each DS and an internal standard containing 50 µg proteins of all five DSs were labelled with fluorescent dye to allow separate detection. The preparative gels were loaded with additional 400 µg of unlabelled protein of the internal standard to enable Coomassie staining and detection in the end. Randomised combination of protein samples and dyes on 2D-DIGE gels 1-15 was as follows (Gel number, dye 1 / dye 2):

- **Gel 1, 6, 11:** DS 1 / DS 3
- **Gel 2, 7, 12:** DS 2 / DS 5
- **Gel 3, 8, 13:** DS 3 / DS 4
- **Gel 4, 9, 14:** DS 5 / DS 2
- **Gel 5, 10, 15:** DS 4 / DS 1

Every separation was performed with three technical replicates.

In the first dimension the proteins were separated on a 24 cm Immobiline DryStrip gel (IGP strip) with nonlinear pH range 3–11 (GE Healthcare Europe GmbH, Freiburg, Germany) using the isoelectric focussing system Ettan IPGphor II (Amersham plc, Little Chalfont, UK) according to the following protocol: 3 h 0-300 V (gradient), 4 h 300-600 V (gradient), 4 h 600-1,000 V (gradient), 4 h 1,000-8,000 V (gradient), 3 h 8,000 V (step). After isoelectric focussing the IPG strips were equilibrated for 15 min in 10 mL of equilibration buffer (6 M urea, 30% glycerol [*v/v*], 2% sodium dodecyl sulphate (SDS) [*w/v*], 75 mM TRIS, 0.002% bromophenol blue [*w/v*]) containing 1% dithiothreitol [*w/v*], and equilibrated subsequently for 15 min in 10 mL of equilibration buffer containing 2.5% iodoacetamide [*w/v*].

For separation of proteins in the second dimension, SDS polyacrylamide gels 12% [*w/v*] of 1.0 mm thickness were casted via the Ettan DALTsix Electrophoresis Unit (GE Healthcare Europe GmbH, Freiburg, Germany). The separation conditions were as follows: 1 W/gel for 1 h followed by 15 W/gel for 5 h. The proteins were visualised with the FLA-9000 image scanner (Fujifilm Life Science, Stamford, CT, USA) using a resolution of 100 µm. Gel images were analysed with Delta2D 4.5.3 software (DECODON GmbH, Greifswald, Germany), using the group warping strategy (default settings) and union fusion with an 86 pixel visual background region of the fusion image. After spot detection (average spot size: 28 pixels, sensitivity: 20) and default normalisation of spot volume, spots showing at least twofold down- or upregulation when comparing DSs 1/3, 2/4, 3/5, and the successive DSs were identified. After removal of doublings and artefacts, 78 spots were chosen. Afterwards, preparative gels were stained with Coomassie Brilliant Blue R250 and visualised by a GS-800 Calibrated Densitometer (BioRad Laboratories GmbH, Feldkirchen, Germany). The spots of interest were cut manually from preparative gels 1 and 8 (third replicate was excluded because of insufficient quality).
